# Examining associations between human milk fatty acids, oligosaccharides, and early infant cognitive, language and motor development in the CHILD cohort study

**DOI:** 10.3389/fnut.2025.1606169

**Published:** 2025-10-01

**Authors:** Sarah E. Turner, Leslie E. Roos, Nathan C. Nickel, Theo J. Moraes, Stuart E. Turvey, Elinor Simons, Padmaja Subbarao, Bianca Robertson, Joyce Chikuma, Susan Goruk, Catherine J. Field, Lars Bode, Jacqueline Pei, Piushkumar J. Mandhane, Meghan B. Azad

**Affiliations:** ^1^Manitoba Interdisciplinary Lactation Centre (MILC), Winnipeg, MB, Canada; ^2^Children’s Hospital Research Institute of Manitoba, Winnipeg, MB, Canada; ^3^Department of Community Health Sciences, University of Manitoba, Winnipeg, MB, Canada; ^4^Manitoba Centre for Health Policy, University of Manitoba, Winnipeg, MB, Canada; ^5^Department of Psychology, University of Manitoba, Winnipeg, MB, Canada; ^6^Faculty of Kinesiology and Recreation Management, University of Manitoba, Winnipeg, MB, Canada; ^7^Department of Pediatrics, The Hospital for Sick Children, University of Toronto, Toronto, ON, Canada; ^8^Department of Pediatrics, University of British Columbia, Vancouver, BC, Canada; ^9^Department of Pediatrics and Child Health, University of Manitoba, Winnipeg, MB, Canada; ^10^Department of Pediatrics, Physiology and Dalla Lana School of Public Health, The Hospital for Sick Children, University of Toronto, Toronto, ON, Canada; ^11^Department of Pediatrics, Larsson-Rosenquist Foundation Mother-Milk-Infant Center of Research Excellence (MOMI CORE), and the Human Milk Institute (HMI), University of California San Diego, San Diego, CA, United States; ^12^Department of Pediatrics, University of Alberta, Edmonton, AB, Canada; ^13^Department of Agriculture, Food and Nutrition, University of Alberta, Edmonton, AB, Canada; ^14^School and Child Psychology Program, University of Alberta, Edmonton, AB, Canada; ^15^Faculty of Medicine and Health Sciences, UCSI University, Kuala Lumpur, Malaysia

**Keywords:** cognitive development, language developement, motor development, breast milk composition, Bayley-III Scales of Infant and Toddler Development, cohort study

## Abstract

**Introduction:**

Human milk fatty acids and human milk oligosaccharides (HMOs) are milk components inconsistently associated with neurodevelopment. The objective of this research is to examine the link between fatty acids, HMOs and neurodevelopment.

**Methods:**

This study includes a subset of 240 parent-infant pairs from the Edmonton site of the CHILD Cohort Study. At 3–4 months post-partum, breastfeeding parents provided a milk sample which was analyzed to identify 20 fatty acids and 19 HMOs. Research assistants administered the Bayley Scales of Infant and Toddler Development at 1 and 2 years of age, comprising cognitive, language and motor development scales (standardized to a mean of 100 and a standard deviation of 15; higher scores indicate better development). Adjusted linear regression was used to estimate the relationships between individual milk components or principal components and neurodevelopment, adjusting for maternal and infant factors. Interactions were tested with infant sex and maternal secretor status.

**Results:**

After adjustment, the first fatty acid principal component, characterized by high saturated fat and low n-3 and n-6 fatty acids, was related to higher motor scores (*β* = 1.59; 95% CI: 0.75, 2.43). Higher concentrations of disialyllacto-N-tetraose were related to lower motor scores (*β* = −3.91, 95% CI: −5.81, −2.01). Higher concentrations of difucosyllacto-N-hexaose were related to higher language and motor scores for infants of maternal non-secretors, while higher concentrations of 3′-sialyllactose were related to higher scores for infants of maternal secretors.

**Conclusion:**

Both fatty acids and HMOs are related to early neurodevelopment. Maternal secretor status moderates the relationship between select HMOs and neurodevelopment.

## Introduction

Decades of research have shown that longer breastfeeding duration and more exclusive breastfeeding are related to better neurodevelopmental outcomes such as performance on intelligence tests and standardized measures of cognitive, language or motor development (1–4). A prominent hypothesis to explain the association between breastfeeding and child neurodevelopment is that human milk contains the optimal source of nutrients (ex. fats, protein, carbohydrates) and other bioactive components (ex. human milk oligosaccharides, brain-derived neurotrophic factor, milk fat globule membrane) to support the developing brain (5, 6). Thus, longer duration, and more exclusive breastfeeding would provide more of these nutritional and bioactive components and confer more benefits to the infant. However, our current understanding of which human milk components contribute to neurodevelopment is limited and requires further study.

### Human milk fatty acids and infant neurodevelopment

Fatty acids are the most well-studied milk component related to neurodevelopment (7). It is well known that both n-3 and n-6 long chain polyunsaturated fatty acids (LCPUFAs) have essential roles in human brain development and function (8, 9); however, the available observational data on the association between human milk LCPUFAs and child neurodevelopment has produced mixed results. Some previous studies have shown that higher concentrations of human milk LCPUFAs, measured within the first 4 months post-partum, are related to better neurodevelopment (i.e., infant temperament and psychomotor development) (10, 11); while others have shown no relationship with neurodevelopment (i.e., cognition and intelligence) (12, 13). Further, it is largely unknown if other human milk fatty acids, such as saturated and monounsaturated fats, are related to child neurodevelopment; this relationship needs to be explored further.

Evidence from rodent and human adult studies have shown sex-specific pathways for fatty acid metabolism (14, 15), and emerging research indicates that concentrations of human milk ALA and total n-3 LCPUFAs measured after 8 weeks post-partum are higher in milk made for female infants compared to males (16). In addition, language scores at 13 months, and cognitive scores at 24 months are known to differ based on infant gender, with girls having higher mean scores than boys (17). This evidence provides rational to study the moderating role of child sex on the association between human milk fatty acids and child neurodevelopment to glean more nuanced and specific understandings of these relationships. In general, further research is needed to clarify the associations between human milk n-3 and n-6 fatty acids and child neurodevelopment, investigate novel associations between saturated and monounsaturated fats and child neurodevelopment, and test for sex differences.

### Human milk oligosaccharides and infant neurodevelopment

Another human milk component that has received attention for its association with infant neurodevelopment are human milk oligosaccharides (HMOs) (18). HMOs are complex, bioactive, sugar molecules that have multiple functions, one of which is to shape the infant gut microbiome, which can modulate infant brain development through the gut-brain axis (19). In addition, sialic acid, one of the HMO building blocks, is essential for human brain ganglioside development which play an important role in cell signaling and communication (20). A recent narrative review identified only five previous human studies in term infants (four of which *n* < 100) examining the association between HMOs and infant neurodevelopment up to 2 years of age (18).

There is some evidence that associations between individual HMOs and child outcomes, including neurodevelopment, may vary depending on maternal secretor status, a genetic trait that has a large influence on the type and number of HMOs produced in human milk (21, 22). Additionally, it is largely unknown if the relationships between HMOs and neurodevelopment are moderated by infant sex. This emerging area of research requires more study to understand the associations between HMOs and child neurodevelopment and the potential moderation by maternal secretor status and infant sex.

### Objectives

The objectives of these exploratory analyses are to 1), examine the associations between 20 fatty acids and 19 HMOs in relation to child cognitive, language and motor development at 1 and 2 years of age, 2), using dimension reduction techniques, assess the association between fatty acids, HMOs and neurodevelopment, and 3), determine if these associations are moderated by sex, or maternal secretor status (HMOs only).

## Methods

### Data and sample

This study uses data from the CHILD cohort study, a pan-Canadian longitudinal cohort ongoing since 2009 (*n* = 3,407) (23). Data were collected in four sites across Canada; Toronto, Manitoba (including Winnipeg, Morden and Winkler), Edmonton and Vancouver, and at several time points (i.e., two pre-natal assessments and annual data collection through a combination of visits and questionnaires). The CHILD study is unique because it combines data from biological samples as well as survey data to better understand relationships between environmental exposures and biological functions. Parent-infant pairs were included if the infant was born at or after 35 weeks gestation, the parent had the ability to read and write English and the pregnant parent was greater than 18 years of age at the time of recruitment. Pairs were excluded if the child was born with major congenital abnormalities, a child of multiple births, a child resulting from *in vitro* fertilization or a child who would not spend at least 80% of nights in the index home (*n* = 111). Written informed consent was obtained from all participants at enrollment. The study was approved by the Human Research Ethics Boards at the University of Alberta, the University of British Columbia, McMaster University and the University of Manitoba.

Parents who were breastfeeding at a mean age of 4 months post-partum (range: <1 month to 11 months) were asked to provide a 10 mL breastmilk sample that consisted of a mix of foremilk and hindmilk from multiple feedings during a 24-h period (*n* = 2,571) (24). Following collection, parents were instructed to place the sample in the refrigerator for up to 24 h until the sample was collected by study staff and placed in a −80 °C freezer. A subset of milk samples was preselected to be analyzed for both fatty acids and HMOs (*n* = 1,200), however, only 1,181 samples were analyzed for both milk components and had corresponding survey data. This subsample was partly representative of the CHILD study population (about 1/3 of the samples) and partly enriched for maternal and infant health conditions (i.e., asthma, allergies and obesity; about 2/3 of the samples). To improve normality of the dataset, we excluded dyads with ≥ one milk fatty acid or oligosaccharide value that was ± > 6 standard deviations (SD) from the mean (*n* = 26). Two further dyads were excluded because the infant had parent-reported, physician diagnosed Trisomy 21. The main outcome of this study, the Bayley Scales of Infant and Toddler Development III (Bayley-III), was only measured in the Edmonton cohort, therefore, 913 dyads from the other three sites were excluded because they did not have Bayley-III data. In total 240 parent–child dyads were included in the current analysis (Supplementary Figure S1).

### Measures

#### Milk fatty acids

Milk fatty acids were analyzed by gas chromatography at the University of Alberta and expressed as relative percentages of total identified fatty acids (25). Relative percentages were used instead of absolute concentrations because the sampling protocol did not involve a full breast expression, which is necessary to accurately determine fat content and concentrations. Relative percentages of milk fatty acids are commonly used in human milk research including previously within the CHILD Study (26–28). All values were *z*-scored with a mean of zero and a standard deviation (SD) of one so the values could be comparable across all fatty acids. Twenty commonly identified fatty acids were included in the present analysis. These included saturated fatty acids [SFA; capric acid (10:0), lauric acid (12:0), myristic acid (14:0), palmitic acid (16:0), margaric acid (17:0) and stearic acid (18:0)], monounsaturated fatty acids [MUFA; palmitoleic acid (16:ln-7), oleic acid (18:ln-9), and vaccenic acid (18:1 c-11)], n-3 polyunsaturated fatty acids [n-3 PUFA; *α*-linoleic acid (ALA; 18:3n-3), eicosatetraenoic acid (ESA; 20:4n-3), eicosapentaenoic acid (EPA; 20:5n-3), docosapentaenoic acid (DPA; 22:5n-3), and docosahexaenoic acid (DHA; 22:6n-3)] and n-6 polyunsaturated fatty acids [n-6 PUFA; linoleic acid (LA; 18:2n-6), *γ*-linolenic acid (GLA; 18:3n-6), conjugated linoleic acid (CLA; 18:2c-9, t-11), dihomo-γ-linoleic acid (DGLA; 20:3n-6), arachidonic acid (ARA; 20:4n-6) and adrenic acid (22,4n-6)]. In addition, similar to previous research, five biologically meaningful ratio variables and four summary variables were created (28). The ratios included: ARA: (DHA + EPA); ARA: DHA; total n-6:total n-3; LA: ALA; (EPA + DPA): DHA (all calculated using non-transformed data), and the summary measures included: total n-3; total n-6; total n-3 without ALA and total n-6 without LA.

#### Human milk oligosaccharides

We identified 19 HMOs using high-throughput solid-phase extraction and analyzed by liquid chromatography at the University of California San Diego. The 19 HMOs identified account for approximately 90% of the total HMO content and were summed to approximate the total HMO content per sample (29). The absolute concentrations of each HMO were log-transformed for normality and then z-score transformed with a mean of zero and a SD of one to be comparable across all HMOs in the sample. The HMOs included fucodisialyllacto-N-hexaose (FDSLNH); 2′-fucosyllactose (2’FL); 3-fucosyllactose (3FL); difucosyllactose (DFLac); lacto-N-fucopentaose-I (LNFP I); lacto-N-fucopentaose-II (LNFP II); lacto-N-fucopentaose-III (LNFP III); difucosyllacto-N-tetrose (DFLNT); fucosyllacto-N-hexaose (FLNH); difucosyllacto-N-hexaose (DFLNH); 3′-sialyllactose (3’SL); 6′-sialyllactose (6’SL); sialyllacto-N-tetraose b (LSTb); sialyllacto-N-tetraose c (LSTc); disialyllacto-N-tetraose (DSLNT); disialyllacto- N-hexaose (DSLNH); lacto-N-neotetraose (LNnT); lacto-N-tetrose (LNT) and lacto-N-hexaose (LNH). In addition, three summary measures were included: total HMO-bound sialic acid, total HMO-bound fucose and total HMOs.

#### Bayley Scales of Infant and Toddler Development III

The Bayley Scales of Infant and Toddler Development III (Bayley-III) was administered to children at 1 and 2 years of age in the Edmonton cohort by trained research assistants (4, 30). The Bayley-III is an assessment that measures developmental functioning of infants and toddlers between 1 and 42 months of age. For the purposes of these analyses, we included three assessed domains: cognitive, language, and motor development. Composite scores for each domain are standardized to a mean of 100 and a SD of 15, with higher scores indicating better development.

#### Maternal secretor status

Maternal secretor status is a genetic trait determined by the fucosyltransferase 2 (FUT2) gene, which in about 20% of the population is inactivated by a single nucleotide polymorphism leading to truncation of the gene product (31). Secretor status impacts the number and type of HMOs that the mother produces, particularly 2’FL which is the most abundant HMO among secretors but is virtually absent among non-secretors (29). Maternal secretor status was determined by the presence or near absence of the 2’FL in the mother’s milk.

#### Confounders

We developed a directed acyclic graph to identify variables to use as confounders in the present analysis (Supplementary Figure S2). These include child sex (male or female), birth mode (cesarian or vaginal), birthweight (continuous in kilograms), gestational age (continuous in weeks), number of older siblings (none, one or two or more), maternal race (White, Asian, or Other), completed maternal post-secondary education (yes/no) and infant age at milk sampling (continuous in weeks). In sex interaction models, child sex was used as a moderator, not as a confounder.

### Statistical analyses

Characteristics of the sample were described and compared to those who had Bayley-III scores at 1 year but no milk component data. Then, we ran Pearson correlations between fatty acids, HMOs and cognitive, language and motor scores at 1 year and 2 years of age. Pearson correlations were used because the milk components and Bayley-III scores generally follow a normal distribution. A sensitivity analysis was conducted repeating the analysis among just the exclusively breastfed infants with the intent of reducing statistical variance in the outcome due to the consumption of infant formula or other food. To understand the shape of the relationships and visually assess the potential impact of outliers, scatter plots were generated displaying the linear, quadradic and cubic fit lines for the one-year Bayley-III outcomes.

Next, to assess for potential confounding, we ran linear regression models between milk components and Bayley-III scores at 1 and 2 years, adjusting for sex, birth mode, birthweight, gestational age, number of older siblings, maternal race, maternal post-secondary degree and infant age at milk sampling. We combined the two milk component types into one analysis to assess their potential interdependence in relation to neurodevelopment using principal component analysis as a dimension reduction technique. We determined the first component that explained the largest amount of variance in the sample of fatty acids and HMOs, separately. Then, we entered both first components in a regression model, with Bayley-III scores at 1 and 2 years at the outcomes, to determine if either milk component accounted for variation in the other component. We also limited the regression models to secretors and non-secretors to determine if there were differences in the associations with Bayley-III scores when looking at these specific subgroups. Sex interactions, adjusting for all confounders, were tested for all fatty acids and HMOs in relation to all Bayley-III scores. Adjusted maternal secretor status interactions were tested for HMOs. All *p*-values throughout the analysis were corrected for multiple comparisons using the Benjamini Hochberg False Discovery Rate (32).

## Results

### Demographics of the sample

Of the 653 infants who had Bayley-III scores at 1 year, 240 were included in the present analysis because they also had an analyzed milk sample (Table 1). At the time of milk sampling, 45.4% of infants were being exclusively breastfed. The mean infant age at milk sampling was 18.1 (± 4.7) weeks. About 72.0% of the mothers who provided milk samples were secretors. The mean Bayley-III scores at 1 year were 110.9 (±10.1) for the cognitive domain, 109.8 (±11.4) for the language domain and 104.7 (±14.5) for the motor domain. At 2 years, the scores were slightly lower with mean scores of 106.9 (± 15.6) for the cognitive domain, 101.2 (±12.2) for the language domain and 99.1 (± 9.7) for the motor domain. Compared to infants in the CHILD study with a Bayley-III assessment but no milk sample (*n* = 413), those with milk samples were more likely to be exclusively breastfed at 3 months and have higher Bayley-III language scores at 1 year (*p*-values ≤0.001 and 0.02, respectively); the other demographics were not statistically different between the subset used for this analysis and all infants with a Bayley-III assessments at 1 year.

**Table 1:** Characteristics of CHILD cohort subset included in the current analysis of fatty acid and HMO data.

Variable	*n* (%) or mean [SD]		
	Subset for this analysis (*n* = 240)	Remaining infants with a Bayley-III score at 1 Year^a^ (*n* = 413)	Chi square or Wilcoxon test *p*-value
Independent variables			
Breastfeeding at 3 months^b^			
None	1 (0.4)	100 (24.4)	≤0.001
Partial	76 (31.7)	99 (24.2)	
Exclusive	163 (67.9)	210 (51.3)	
Breastfeeding status at time of milk sampling			
Partial	124 (54.6)	-	
Exclusive	103 (45.4)	-	
Maternal secretor status			
Non-secretor	68(28.3)	-	
Secretor	172 (71.7)	-	
Child sex			
Female	119 (49.6)	205 (49.6)	0.99
Male	121 (50.4)	208 (50.4)	
Bayley scales 1 year			
Cognitive	110.9 [10.1]	109.7 [10.4]	0.10
Language	109.8 [11.4]	107.3 [12.8]	0.02
Motor	104.7 [14.5]	103.0 [14.1]	0.20
Bayley scales 2 year			
Cognitive	106.9 [15.6]	104.8 [13.8]	0.14
Language	101.2 [12.2]	99.3 [12.2]	0.16
Motor	99.1 [9.7]	98.6 [10.0]	
Confounders			
Maternal race			
White	187 (78.2)	316 (77.8)	0.98
Asian	28 (11.7)	47 (11.6)	
Other	24 (10.0)	43 (10.6)	
Maternal education^c^			
Some or Completed high school	14 (6.0)	35 (8.9)	0.10
Some college or University	36 (15.5)	80 (20.3)	
Completed college or University	183 (78.5)	279 (70.8)	
Birth mode			
Vaginal	175 (73.8)	315 (76.6)	0.42
C-section	62 (26.2)	96 (23.4)	
Birthweight (kg)	3.5 [0.5]	3.4 [0.5]	0.13
Gestational age (weeks)	39.0 [1.4]	39.1 [1.4]	0.52
Older siblings			
None	125 (52.1)	176 (42.6)	0.06
One	84 (35.0)	176 (42.6)	
Two or more	31 (12.9)	61 (14.8)	
Infant age at milk sampling (weeks)	18.1 [4.7]	-	

Chi-square test of independence is used to test differences between categorical variables. Wilcoxon Rank Sum Test is used to test differences between continuous variables. ^a^ Infants with a Bayley-III (Bayley Scales of Infant and Toddler Development) score and no fatty acid or HMO data. ^b^ One parent provided a milk sample before they responded to the three-month questionnaire, at which point they had stopped breastfeeding. ^c^ Maternal education is collapsed into two categories (less than post-secondary education and post-secondary education) when used as a confounder. HMOs, Human Milk Oligosaccharides; SD, standard deviation.

### Select fatty acids and HMOs are related to cognitive, language and motor development at 1 year

**Figure 1:**
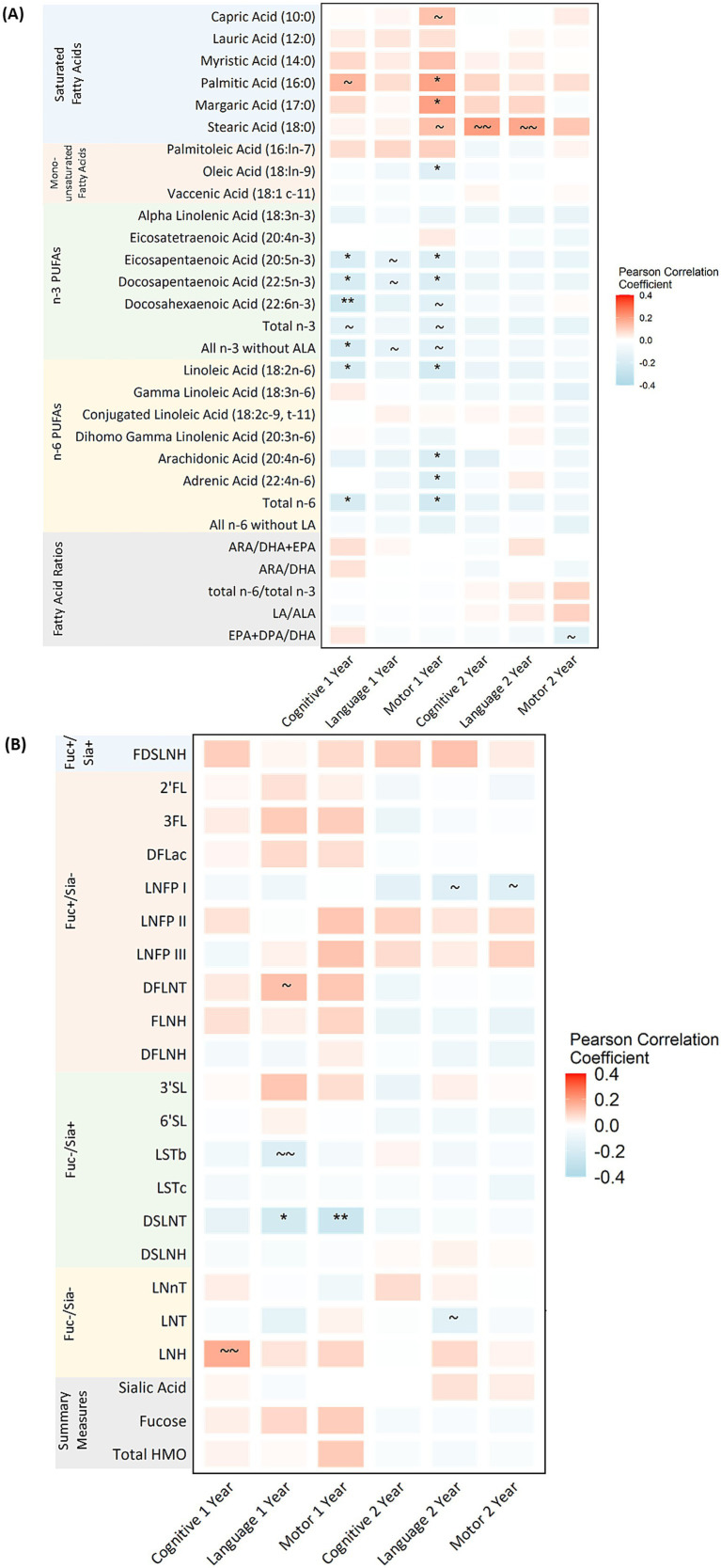
Pearson correlations between human milk fatty acids **(A)**, HMOs **(B)** and Bayley-III scores at 1 and 2 years of age in the CHILD cohort study. Bayley-III, Bayley Scales of Infant and Toddler Development; DFLac, difucosyllactose; DFLNH, difucosyllacto-N-hexaose; DFLNT, difucosyllacto-N-tetrose; DSLNH, disialyllacto- N-hexaose; DSLNT, disialyllacto-N-tetraose; FLNH, fucosyllacto-N-hexaose; FDSLNH, fucodisialyllacto-N-hexaose; Fuc, Fucosylated HMO; Fucose, human milk oligosaccharide–bound fucose; HMO, human milk oligosaccharide; LNFP I/II/III, lacto-N-fucopentaose-I/II/III; LNH, lacto-N-hexaose; LNnT, lacto-N-neotetraose; LNT, lacto-N-tetrose; LSTb/c, sialyllacto-N-tetraose b/c; Sia, Sialylated HMO; Sialic Acid, HMO-bound Sialic Acid; 2’FL, 2′-fucosyllactose; 3FL, 3-fucosyllactose; 3’SL, 3′-sialyllactose; 6’SL, 6′-sialyllactose; PUFA, Polyunsaturated Fatty Acid; ALA, Alpha Linolenic Acid; EPA, Eicosapentaenoic Acid; DPA, Docosapentaenoic Acid; DHA, Docosahexaenoic Acid; LA, Linoleic Acid; ARA, Arachidonic Acid. All milk components are expressed as *z*-scores aside from fatty acid ratios which are not z-scored. See Supplementary Figure S3 for exact correlation coefficients. See Supplementary Figures S4, S5 for a sensitivity analysis limited to exclusively breastfed infants and Supplementary Figures S6, S7 for scatter plots indicating linear and polynomial fit lines. *FDR corrected *p*-value ≤0.05, ** FDR corrected *p*-value ≤0.01, ~ uncorrected *p*-value ≤0.05, ~ ~ uncorrected *p*-values ≤0.01.

There were significant correlations between select fatty acids and cognitive and motor development at 1 year of age after FDR adjustment (Figure 1A; Supplementary Figure S3A). Specifically, higher proportions of n-3 and n-6 PUFAs including, EPA (20:5n-3), DPA (22:5n-3), DHA (22:6n-3), total n-3 without ALA, LA (18:2n-6) and total n-6 fatty acids were related to lower (worse) cognitive scores (rho range from −0.24 to −0.18). These correlations generally became stronger when limiting to those who were exclusively breastfed (rho range from –0.36 to −0.28; Supplementary Figures S4A, S5A). Two saturated fatty acids were related to higher (better) motor development at 1 year: palmitic acid (16:0) and margaric acid (17:0) (rho = 0.19 and 0.20, respectively). In addition, several monounsaturated, n-3 and n-6 PUFAs were related to lower (worse) motor development at 1 year including: oleic acid (18:ln-9), EPA (20:5n-3), DPA (22:5n-3), LA (18:2n-6), ARA (20:4n-6), AA (22:4n-6) and total n-6 (rho range from −0.22 to −0.16). These correlations followed a similar trend when limiting to those who were exclusively breastfed, but none reached statistical significance after FDR correction. After FDR correction, no significant correlations were observed between fatty acids and language development at 1 year or any Bayley-III scales at 2 years and these trends remained when limiting to those who were exclusively breastfed.

Only one HMO was significantly correlated with Bayley-III scores after FDR adjustment; higher DSLNT concentrations were related to lower (worse) language and motor scores at 1 year of age (rho = −0.22 for language and −0.26 for motor; Figure 1B; Supplementary Figure S3B). When limiting to those being exclusively breastfed, there were no significant correlations with one-year outcomes; however, LNFP1 was negatively correlated to motor development at 2 years (rho = −0.39; Supplementary Figures S4B, S5B).

To understand if the relationships between milk components and Bayley-III scores at 1 year followed a linear relationship, we present scatter plots with the linear, quadradic and cubic (i.e., polynomial) fit lines for fatty acids and HMOs. There were no obvious polynomial fit lines for fatty acids or HMOs and Bayley-III scores at 1 year (Supplementary Figures S6A–C, S7A–C), suggesting generally linear relationships.

### DSLNT remains significantly associated with motor development after adjusting for confounders

**Figure 2:**
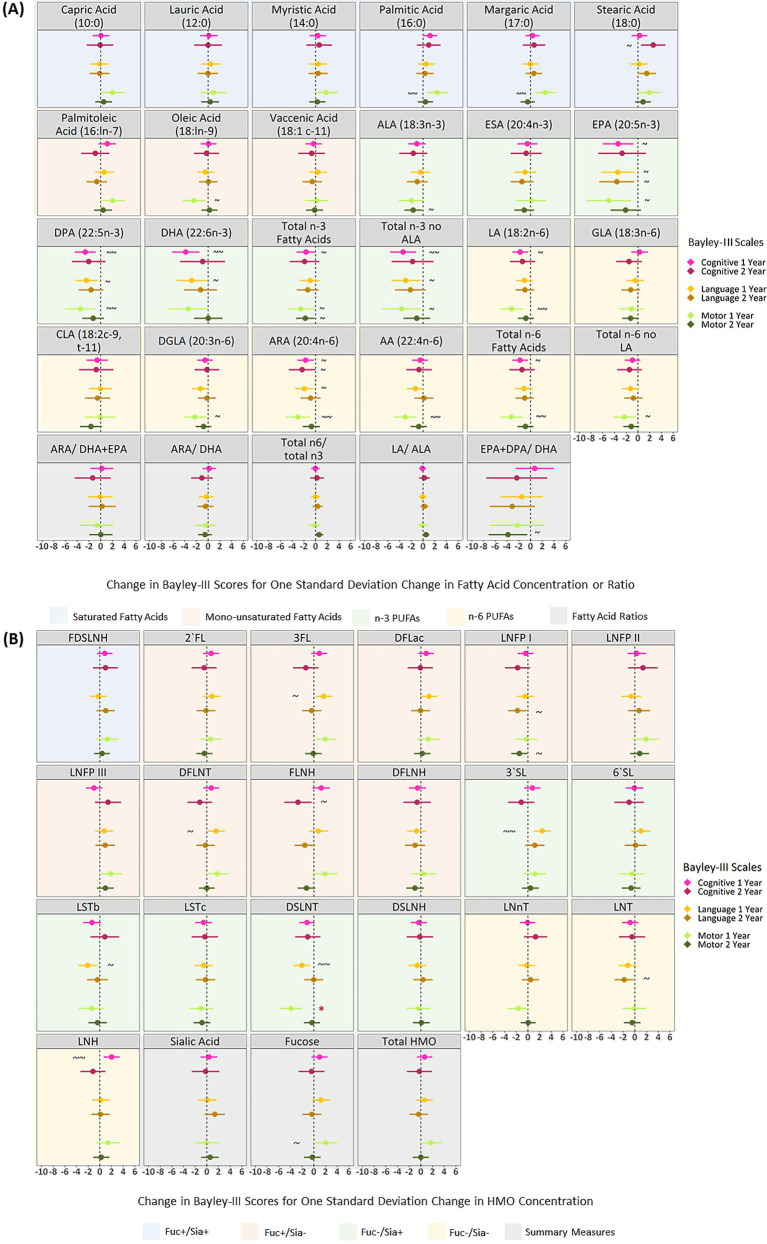
Adjusted linear associations between human milk fatty acids **(A)**, HMOs **(B)** and Bayley-III scores at 1 and 2 years of age in the CHILD cohort study. All models are adjusted for: infant sex, birthweight, birth mode, number of older siblings, gestational age, maternal race, maternal education, infant age at milk sampling. Bayley-III, Bayley Scales of Infant and Toddler Development; DFLac, difucosyllactose; DFLNH, difucosyllacto-N-hexaose; DFLNT, difucosyllacto-N-tetrose; DSLNH, disialyllacto- N-hexaose; DSLNT, disialyllacto-N-tetraose; FLNH, fucosyllacto-N-hexaose; FDSLNH, fucodisialyllacto-N-hexaose; Fuc, Fucosylated HMO; Fucose, human milk oligosaccharide–bound fucose; HMO, human milk oligosaccharide; LNFP I/II/III, lacto-N-fucopentaose-I/II/III; LNH, lacto-N-hexaose; LNnT, lacto-N-neotetraose; LNT, lacto-N-tetrose; LSTb/c, sialyllacto-N-tetraose b/c; Sia, Sialylated HMO; Sialic Acid, HMO-bound Sialic Acid; 2’FL, 2′-fucosyllactose; 3FL, 3-fucosyllactose; 3’SL, 3′-sialyllactose; 6’SL, 6′-sialyllactose; PUFA, Polyunsaturated Fatty Acid; ALA, Alpha Linolenic Acid; ESA, Eicosatetraenoic Acid; EPA, Eicosapentaenoic Acid; DPA, Docosapentaenoic Acid; DHA, Docosahexaenoic Acid; LA, Linoleic Acid; GLA, Gamma Linoleic Acid; CLA, Conjugated Linoleic Acid; DGLA, Dihomo Gamma Linolenic Acid; ARA, Arachidonic Acid; AA, Adrenic Acid. All milk components are expressed at *z*-scores aside from fatty acid ratios which are not *z*-scored. *FDR corrected *p*-value ≤0.05, ~ uncorrected *p*-value ≤0.05, ~ ~ uncorrected *p*-value ≤0.01.

After adjusting for sex, birth mode, birthweight, gestational age, number of older siblings, maternal race, maternal post-secondary degree and infant age at milk sampling, and applying FDR correction, there were no significant associations between human milk fatty acids and Bayley-III scores at 1 or 2 years (Figure 2A). DSLNT remained significantly associated with lower motor scores at 1 year after confounder adjustment and FDR correction (B = −3.91, 95% CI: −5.81, −2.01; i.e. one SD increase in DSLNT is related to a 3.91 point (about ¼ of a SD) decrease in motor scores), however, no other HMOs were significantly related to Bayley-III scores at 1 or 2 years (Figure 2B).

### The first fatty acid principal component is related to motor development at 1 year and this association is unchanged after accounting for HMOs

**Figure 3:**
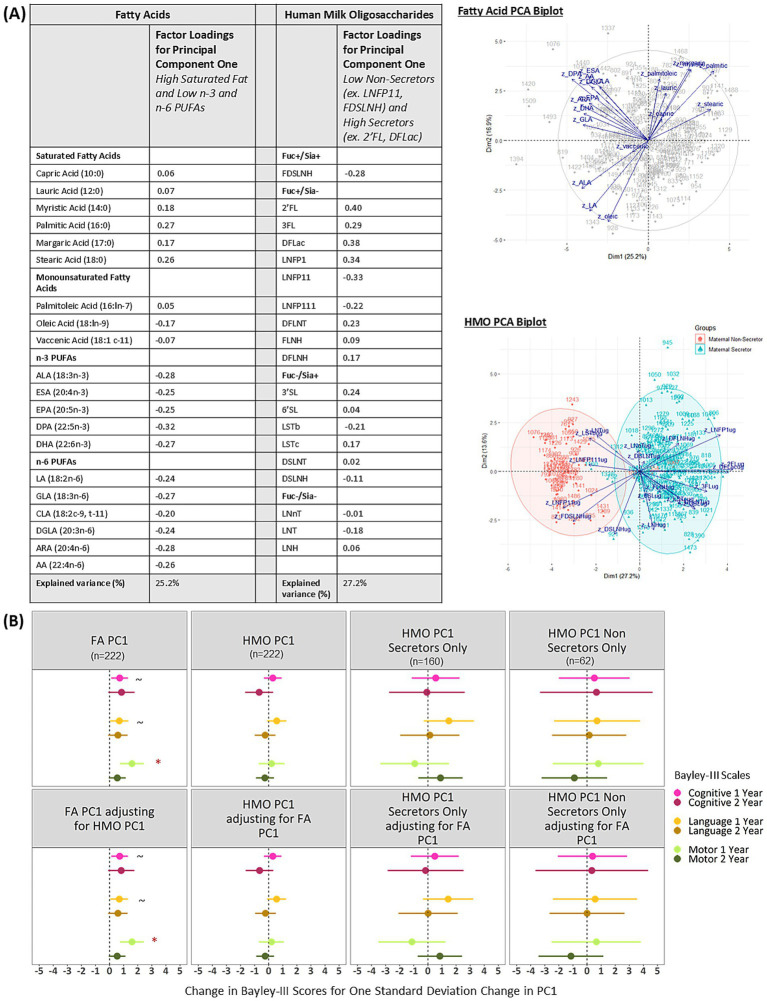
**(A)** Factor loadings and **(B)** Adjusted associations between fatty acid and HMO principal components and Bayley-III scores at 1 and 2 years in the CHILD cohort study. FA PC1 is significantly associated with Bayley-III scores and these relationships are not dependent on HMO PC1. HMO PC1 is not significantly associated with Bayley-III scores; estimates are similar among secretors and non-secretors. Regression models are adjusted for: infant sex, birthweight, birth mode, number of older siblings, gestational age, maternal race, maternal education, infant age at milk sampling. PC, Principal Component; Bayley-III, Bayley Scales of Infant and Toddler Development; DFLac, difucosyllactose; DFLNH, difucosyllacto-N-hexaose; DFLNT, difucosyllacto-N-tetrose; DSLNH, disialyllacto- N-hexaose; DSLNT, disialyllacto-N-tetraose; FLNH, fucosyllacto-N-hexaose; FDSLNH, fucodisialyllacto-N-hexaose; Fuc, Fucosylated HMO; Fucose, human milk oligosaccharide–bound fucose; HMO, human milk oligosaccharide; LNFP I/II/III, lacto-N-fucopentaose-I/II/III; LNH, lacto-N-hexaose; LNnT, lacto-N-neotetraose; LNT, lacto-N-tetrose; LSTb/c, sialyllacto-N-tetraose b/c; Sia, Sialylated HMO; Sialic Acid, HMO-bound Sialic Acid; 2’FL, 2′-fucosyllactose; 3FL, 3-fucosyllactose; 3’SL, 3′-sialyllactose; 6’SL, 6′-sialyllactose; PUFA, Polyunsaturated Fatty Acid; ALA, Alpha Linolenic Acid; ESA, Eicosatetraenoic Acid; EPA, Eicosapentaenoic Acid; DPA, Docosapentaenoic Acid; DHA, Docosahexaenoic Acid; LA, Linoleic Acid; GLA, Gamma Linoleic Acid; CLA, Conjugated Linoleic Acid; DGLA, Dihomo Gamma Linolenic Acid; ARA, Arachidonic Acid; AA, Adrenic Acid. See Supplementary Figure S8 for factor loadings for FA and HMO PC2 and PC3. *FDR corrected *p*-value ≤0.05, ~ uncorrected *p*-value ≤0.05.

Figure 3A shows the factor loadings and biplots for the first fatty acid and HMO principal components (PC1). PC1 explained 25.2% of the variance in the fatty acids data and 27.2% of the variance in the HMO data, which indicates how much information is preserved from the full dataset. Based on the loadings, the PC1s can be described as high saturated fat and low n-3 and n-6 PUFAs and high secretors (ex. 2’FL, DLFac) and low non-secretors (ex. LNFP11, FDSLNH). After adjusting for all confounders and applying the FDR correction, fatty acid PC1 was associated with higher motor scores at 1 year, and this association was unchanged after adjusting for HMO PC1 (B = 1.59; 95% CI: 0.75, 2.43; Figure 3B). Fatty acid PC1 was not significantly associated with Bayley-III scores at 2 years. HMO PC1 was not associated with Bayley-III scores at 1 or 2 years, and the estimates were relatively unchanged after adjusting for FA PC1. In line with the full sample, when limiting to subgroups of either secretors or non-secretors, there were no significant associations between HMO PC1 and Bayley-III scores at 1 or 2 years.

### Maternal secretor status moderates the association between select HMOs and language and motor development at 1 year

**Figure 4:**
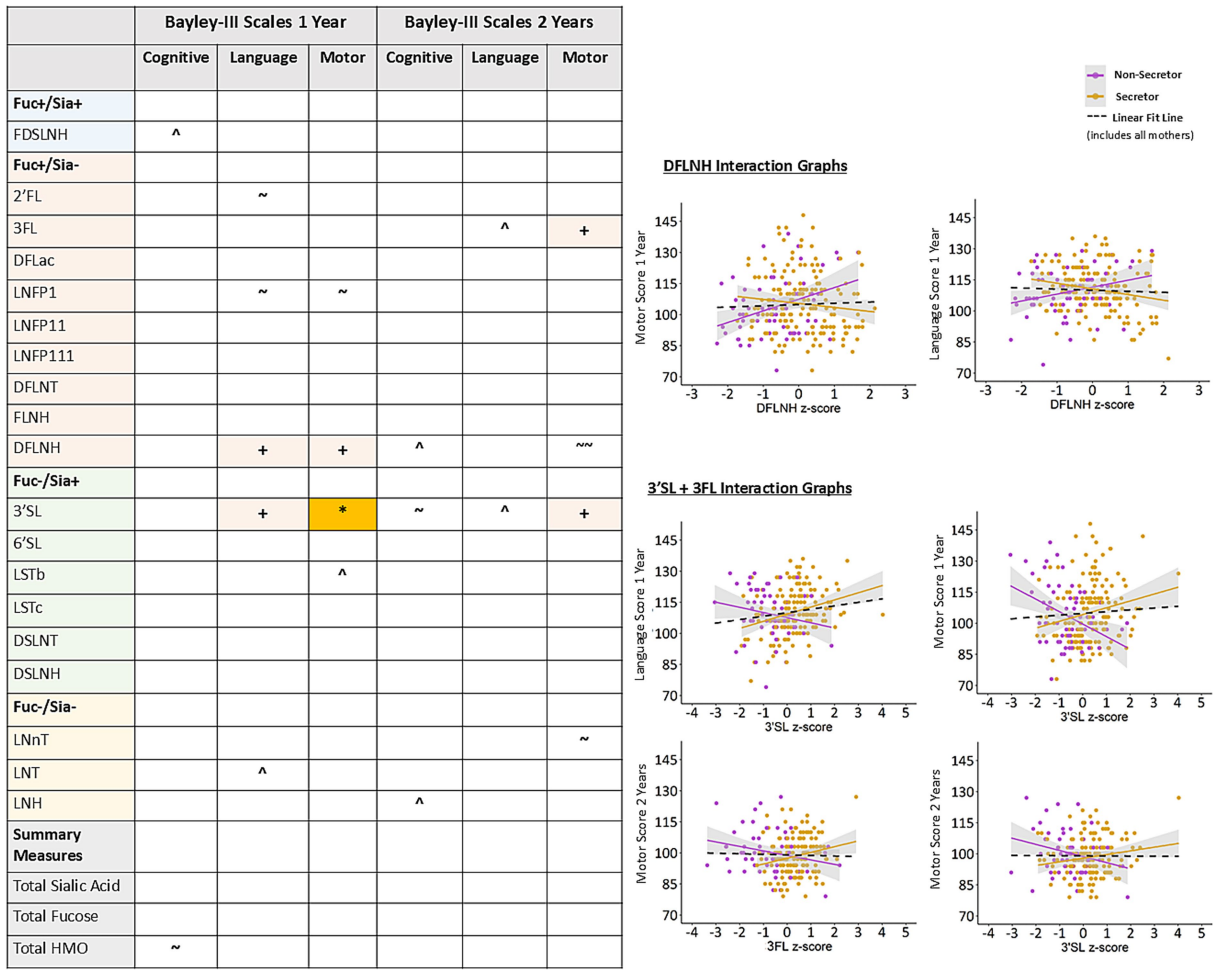
Adjusted interactions between maternal secretor status and HMOs on Bayley-III scores at 1 and 2 years in the CHILD cohort study. Colored cells in the table are represented as scatter plots. All models are adjusted for: infant sex, birthweight, birth mode, number of older siblings, gestational age, maternal race, maternal education, infant age at milk sampling. Bayley-III, Bayley Scales of Infant and Toddler Development DFLac, difucosyllactose; DFLNH, difucosyllacto-N-hexaose; DFLNT, difucosyllacto-N-tetrose; DSLNH, disialyllacto- N-hexaose; DSLNT, disialyllacto-N-tetraose; FLNH, fucosyllacto-N-hexaose; FDSLNH, fucodisialyllacto-N-hexaose; Fuc, Fucosylated HMO; Fucose, human milk oligosaccharide–bound fucose; HMO, human milk oligosaccharide; LNFP I/II/III, lacto-N-fucopentaose-I/II/III; LNH, lacto-N-hexaose; LNnT, lacto-N-neotetraose; LNT, lacto-N-tetrose; LSTb/c, sialyllacto-N-tetraose b/c; Sia, Sialylated HMO; Sialic Acid, HMO-bound Sialic Acid; 2’FL, 2′-fucosyllactose; 3FL, 3-fucosyllactose; 3’SL, 3′-sialyllactose; 6’SL, 6′-sialyllactose. Linear fit line is from the full model that does not include the interaction term in Figure 2B. + FDR corrected *p*-value ≤0.1, *FDR corrected *p*-value ≤0.05, ^ uncorrected *p*-value ≤0.1, ~ uncorrected *p*-value ≤0.05, ~ ~ uncorrected *p*-value ≤0.01.

There were limited adjusted sex interactions in the relationships between milk fatty acids, HMOs and Bayley-III scores at 1 or 2 years of age and none reached statistical significance after confounder adjustment and FDR correction (Supplementary Figures S9, S10). Maternal secretor status interactions showed that DFLNH was significantly related to better language and motor scores at 1 year for infants of maternal non-secretors, while 3’SL was related to better language and motor scores at 1 year for infants of maternal secretors (Figure 4). Significant interactions between 3’SL, 3FL and secretor status also showed better motor scores at 2 years for infants of maternal secretors. These results indicate that maternal secretor status may play an important role in moderating the relationships between DFLNH, 3’SL, 3FL, and language and motor development, however, the direction of the moderation effect is not consistent (i.e., having a positive secretor status is not always better).

## Discussion

This is the largest study to date to examine relationships between both human milk fatty acids and HMOs with child neurodevelopment at ages 1 and 2 years. Our results suggest that select n-3 and n-6 PUFAs were related to lower cognitive and motor scores at 1 year of age, while select saturated fatty acids were related to higher motor scores. Although these individual associations did not withstand correction for multiple comparisons, they were corroborated by results from principal component analyses where fatty acid profiles high in saturated fat and low in n-3 and n-6 PUFAs were significantly related to better motor scores at 1 year. Higher concentrations of DSLNT, a sialylated HMO, were related to lower motor scores at 1 year and this association was robust to confounding and correction for multiple comparisons. However, overall HMO profiles were not associated with neurodevelopment at 1 or 2 years. We found that secretor status moderates associations between DFLNH, 3’SL, 3FL and language and motor scores, however, the direction of the moderation effect was inconsistent between these HMOs.

### Select n-3 and n-6 PUFAs are related lower cognitive and motor development scores at 1 year

Contrary to the existing literature (10, 11, 33), in unadjusted models, higher concentrations of n-3 PUFAs were related to *worse* cognitive and motor scores at 1 year. There is existing research showing no association between n-3 PUFAs and neurodevelopment both in observational studies of human milk n-3 PUFAs and RCTs of n-3 formula supplementation (7, 34); however, to our knowledge, no studies have shown negative associations between n-3 PUFAs and neurodevelopment in term infants. A previous analysis using data from the CHILD cohort found that DHA was negatively associated with head circumference, a proxy for brain development (35), which may also be related to cognitive, language and motor scores (36). While the associations shown in the current study did not persist after adjustment and FDR correction, or extend to the two-year time point, the direction of the associations are consistently negative across most n-3 and n-6 PUFAs. It is important to note that the relative proportion of fatty acids in the milk does not reflect the actual amount of fatty acid that gets absorbed and utilized by the infant body, or how absorption is affected by the presence of other milk components. These cautions may shed light on the reasons for our negative findings. Future research could examine infant serum to determine absorbed fatty acids levels and how this may be associated with Bayley-III scores. Additionally, future work is needed to examine different combinations of human milk components to determine if and how their bioavailability interacts in the infant body.

### Saturated fatty acids are related to better motor development at 1 year

This is one of the first studies to examine the relationship between human milk saturated fatty acids and child neurodevelopment. Palmitic and margaric acid were related to better motor scores at 1 year in unadjusted models, although the associations did not remain significant in adjusted models and after FDR correction. However, the fatty acid profile high in saturated fat and low n-3 and n-6 PUFAs (the first principal component) was related to better motor scores at 1 year and this association was robust to confounding and FDR correction. Principal component analysis preserves variance which may translate into increased statistical power to observe a significant effect compared to individual analyses (37).

Saturated fats comprise the largest proportion of milk fat [42.2% in mature milk (38)] and can help to absorb fat-soluble vitamins and assist other biological functions, however, most research on the health effects of saturated fat has been in older children or adults (39, 40). Typically, increased dietary saturated fat is related to poorer health outcomes, including lower cognitive flexibility in children ages 7 to 10 years old (39). The results from the current study suggest that human milk saturated fatty acids may play a positive role in infant neurodevelopment but requires replication in other cohorts.

### DSLNT is related to lower motor development scores at 1 year

Two previous human studies have examined the relationship between DSLNT and motor development and results were counter to what was observed in the current analysis. Both Ferrira et al. (41) and Sato et al. (42) showed that higher concentrations of DSLNT were related to better fine motor development, as measured by the Ages and Stages Questionnaire, in infants at 6 months and 1 year of age. However, in alignment with the current study, the association between DSLNT and motor development did not persist to the two-year time point in Sato et al. (42). Differences in the timing of human milk sample collection (i.e., at 1 month in both Ferrira et al. (41) and Sato et al. (42), while mean sample collection was 4 months in the current study), or the neurodevelopment measure, could explain the diverging findings.

It is currently understood that the primary role of HMOs is to act as prebiotics for gut bacteria and that certain HMOs have strong effects on shaping the infant microbiome (43). One study showed that DSLNT was related to multiple different bacterial genera in the infant gut, including a negative association with *Bacteroides* (44). Previous research using the CHILD study data has linked higher abundance of *Bacteroides* with improved motor development (4). Therefore, it is possible that higher concentrations of DSLNT could be linked to lower motor development scores through the reduction of *Bacteroides* in the infant gut.

It is important to note that the main clinical utility of the Bayley-III scale is to assess development and identify children who may not be on a typical developmental trajectory (30). However, the Bayley-III is also used for population-level research, which may result in small effect sizes that can have population-level relevance despite not having individual clinical meaning, as observed in the current study. As explained by Roses Theorem, small effect sizes can still be interpreted as meaningful on a population level because large number of people at a small risk may result in more incidence of the outcome than a small number who are at high risk (45). Infant feeding is a universal exposure, and in Canada, 69% of infants receive some human milk for the first 6 months of life, indicating widespread human milk exposure (46). An alternative method for interpreting the relevance of a novel exposure (e.g., DSLNT in human milk) is to compare the adjusted effect size to that of another variable already known to be associated with that same outcome (i.e., improved neurodevelopment), such as high maternal post-secondary education (47). In our analysis, completing maternal post-secondary education, compared to less than a college or university degree, was related to a 0.33 point increase in Bayley-III motor scores at 1 year (not statistically significant), while a one standard deviation increase in DSLNT was related to a 3.91 point decrease in Bayley-III motor scores (FDR-corrected *p* ≤ 0.05). This demonstrates that, in our sample, DSLNT has an even stronger association with motor development at 1 year than high maternal education and gives context to our findings.

### Fatty acid and HMO principal components are not interdependent

We did not observe interdependent effects of fatty acids and HMO profiles on neurodevelopment. In fact, the significant association between fatty acid PC1 and motor development was unchanged after adjusting for HMO PC1, and similarly, the non-significant associations between HMO PC1 and neurodevelopment were unchanged after adjusting for fatty acid PC1. Previous research has examined combinations of other human milk components (ex. phospholipids, choline and fatty acids) in relation to recognition memory or cognitive development and has found both joint and synergistic associations between milk components (48, 49). To more comprehensively understand relationships between fatty acids and HMOs on neurodevelopment, future work could use more advanced methods such as machine learning or clustering analyses.

### Maternal secretor status moderates the relationship between select HMOs and language and motor scores

The current results align with one previous study that examined differences between HMOs and neurodevelopment based on secretor status. Similar to our findings, Cho et al. (21), found that 3’SL was related to better language development in children between the ages of 2 and 25 months who were born to maternal secretors, with no relationship among children of maternal non-secretors. No other studies have shown associations between DFLNH and higher language and motor scores among infants of maternal non-secretors. While the exact mechanisms of these interactions are unknown, it is possible that certain HMOs have stronger biological effects in a microbial environment that is characterized by secretor status. Typically, infants of maternal secretors have higher abundance of gut Bifidobacteria (50). It is possible that the biological effects of DFLNH are more available in an infant gut that is colonized by bacteria informed by non-secretor milk (ex. less Bifidobacteria). More research in this area may discover neurodevelopmental benefits for infants of maternal non-secretors as well as for infants of maternal secretors.

### Limitations

The results of this exploratory study should be interpreted in the context of several limitations. First, the sampling protocol for milk collection instructed mothers to collect a combination of fore and hind milk from multiple feeds over 24 h and only one milk sample was collected. Concentrations of human milk components can change throughout lactation and concentrations of fatty acids can vary from day to day, throughout a single day, and from the beginning to the end of a feed. However, concentrations of HMOs appear to be stable over 6 h and 7 day periods (51). The current sampling protocol was not designed to capture temporal changes in milk components, and could obscure relevant associations and/or limit the real world-applicability of the results, especially for fatty acids. The CHILD study also did not collect a full breast expression which means the total fat volume in a feed, and therefore the absolute concentrations of fatty acids, cannot be determined. Second, maternal secretor status was phenotypically determined and there is a small chance that genetically determined secretor status could provide different results. Third, CHILD study participants without milk component data were excluded from the analysis; however, there were minimal differences between those included and excluded from the study sample on most variables aside from language scores at 1 year where included infants had higher scores than excluded infants. Therefore, those with lower language scores are underrepresented in this analysis which may underestimate the association between milk components and language scores at 1 year. Fourth, the CHILD study cohort has a higher income, education, and prevalence of married or cohabiting parents than participants in a general population, representative Canadian sample (52). Therefore, the results of this study are not fully generalizable to the general Canadian population.

## Conclusion

In this longitudinal, exploratory study, our results suggest that fatty acid principal component one (comprised of high saturated fat and low n-3 and n-6 PUFAs) is related to higher motor scores at 1 year of age. Additionally, DSLNT, a sialylated HMO, may be related to lower motor scores at 1 year of age. Maternal secretor status may moderate associations between 3FL, 3’SL, DFLNH, and infant language and motor scores; however, positive secretor status did not consistently provide benefits. Results from this work can inform future studies seeking to understand the mechanisms of fatty acids and HMOs on infant neurodevelopment.

## Data Availability

The datasets presented in this article are not readily available because the data requires approval from the CHILD National Coordinating Centre (NCC) before they can be accessed. Requests to access the datasets should be directed to the CHILD NCC to discuss their needs before initiating a formal request. More information about data access for the CHILD Cohort Study can be found at https://childstudy.ca/for-researchers/data-access/. Requests to access these datasets should be directed to child@mcmaster.ca.

## References

[1] Horta BL Victora CG. (2013). Long-term effects of breastfeeding: a systematic review. Available online at: https://www.who.int/publications/i/item/9789241505307 (Accessed March 5, 2025).

[2] McGowan C Bland R. The benefits of breastfeeding on child intelligence, behavior, and executive function: a review of recent evidence. Breastfeed Med. (2023) 18:172–87. doi: 10.1089/bfm.2022.0192, PMID: 36749962

[3] Horta B Bahl R Martines J Victora C. (2007). Evidence on the long-term effects of breastfeeding: systematic reviews and meta-analyses. Available online at: https://iris.who.int/bitstream/handle/10665/43623/9789241595230_eng.pdf (Accessed March 5, 2025).

[4] Tamana SK Tun HM Konya T Chari RS Field CJ Guttman DS. Bacteroides-dominant gut microbiome of late infancy is associated with enhanced neurodevelopment. Gut Microbes. (2021) 13:1–17. doi: 10.1080/19490976.2021.1930875, PMID: 34132157 PMC8210878

[5] Kim SY Yi DY. Components of human breast milk: from macronutrient to microbiome and microRNA. Clin Exp Pediatr. (2020) 63:301–9. doi: 10.3345/cep.2020.00059, PMID: 32252145 PMC7402982

[6] Peila C Riboldi L Coscia A. Role of the biological active components of human milk on long-term growth and neurodevelopmental outcome. Ital J Pediatr. (2024) 50:201. doi: 10.1186/s13052-024-01773-z, PMID: 39350308 PMC11443780

[7] Mitguard S Doucette O Miklavcic J. Human milk polyunsaturated fatty acids are related to neurodevelopmental, anthropometric, and allergic outcomes in early life: a systematic review. J Dev Orig Health Dis. (2023) 14:763–72. doi: 10.1017/S2040174423000454, PMID: 38254254

[8] Sherzai D Moness R Sherzai S Sherzai A. A systematic review of omega-3 fatty acid consumption and cognitive outcomes in neurodevelopment. Am J Lifestyle Med. (2023) 17:649–85. doi: 10.1177/15598276221116052, PMID: 37711355 PMC10498982

[9] Hadley KB Ryan AS Forsyth S Gautier S Salem N. The essentiality of arachidonic acid in infant development. Nutrients. (2016) 8:216. doi: 10.3390/nu8040216, PMID: 27077882 PMC4848685

[10] Hahn-Holbrook J Fish A Glynn LM. Human milk omega-3 fatty acid composition is associated with infant temperament. Nutrients. (2019) 11:1–12. doi: 10.3390/nu11122964 PMC6949911 31817237

[11] Zielinska M Hamulka J Grabowicz-Chądrzyńska I Bryś J Wesolowska A. Association between breastmilk LC PUFA, carotenoids and psychomotor development of exclusively breastfed infants. Int J Environ Res Public Health. (2019) 16:1144. doi: 10.3390/ijerph16071144, PMID: 30935000 PMC6479893

[12] Keim S Daniels J Siega-Riz AM Herring AH Dole N Scheidt PC. Breastfeeding and long-chain polyunsaturated fatty acid intake in the first four post-natal months and infant cognitive development: an observational study. Matern Child Nutr. (2012) 8:471–82. doi: 10.1038/jid.2014.371 21615865 PMC3617566

[13] Gustafsson PA Duchén K Birberg U Karlsson T. Breastfeeding, very long polyunsaturated fatty acids (PUFA) and IQ at 6/4 years of age. Acta Paediatr. (2004) 93:1280–7. doi: 10.1111/j.1651-2227.2004.tb02924.x, PMID: 15499945

[14] Lohner S Fekete K Marosvölgyi T Decsi T. Gender differences in the long-chain polyunsaturated fatty acid status: systematic review of 51 publications. Ann Nutr Metab. (2013) 62:98–112. doi: 10.1159/000345599, PMID: 23327902

[15] Christiansen K Gan M Holman R. Sex differences in the metabolism of fatty acids in virto. Biochim Biophys Acta. (1969) 187:19–25. doi: 10.1016/0005-2795(69)90136-6 5811210

[16] Meng F Uniacke-lowe T Lanfranchi E Meehan G O'Shea CA Dennehy T. A longitudinal study of fatty acid profiles, macronutrient levels, and plasmin activity in human milk. Front Nutr. (2023) 10:2613. doi: 10.3389/fnut.2023.1172613, PMID: 37229467 PMC10203173

[17] Krogh MT Væver MS. Does gender affect Bayley-III scores and test-taking behavior? Infant Behav Dev. (2019) 57:101352. doi: 10.1016/j.infbeh.2019.101352, PMID: 31445432

[18] Berger PK Ong ML Bode L Belfort MB. Human milk oligosaccharides and infant neurodevelopment: a narrative review. Nutrients. (2023) 15:1–10. doi: 10.3390/nu15030719 PMC9918893 36771425

[19] Carlson AL Xia K Azcarate-Peril MA Goldman BD Ahn M Styner MA. Infant gut microbiome associated with cognitive development. Biol Psychiatry. (2018) 83:148–59. doi: 10.1016/j.biopsych.2017.06.021, PMID: 28793975 PMC5724966

[20] Wang B. Molecular mechanism underlying sialic acid as an essential nutrient for brain development and cognition. Adv Nutr. (2012) 3:465S–72S. doi: 10.3945/an.112.001875, PMID: 22585926 PMC3649484

[21] Cho S Zhu Z Li T Baluyot K Howell BR Hazlett HC. Human milk 3′ -sialyllactose is positively associated with language development during infancy. Am J Clin Nutr. (2021) 114:1–10. doi: 10.1093/ajcn/nqab103 34020453 PMC8326052

[22] Jorgensen JM Young R Ashorn P Ashorn U Chaima D Davis JCC. Associations of human milk oligosaccharides and bioactive proteins with infant growth and development among Malawian mother-infant dyads. Am J Clin Nutr. 113:209–20. doi: 10.1093/ajcn/nqaa272, PMID: 33096556 PMC7779225

[23] Subbarao P Anand SS Becker AB Befus AD Brauer M Brook JR. The Canadian healthy infant longitudinal development (CHILD) study: examining developmental origins of allergy and asthma. Thorax. (2015) 70:998–1000. doi: 10.1136/thoraxjnl-2015-207246, PMID: 26069286

[24] Moraes TJ Lefebvre DL Chooniedass R Becker AB Brook JR Denburg J. The Canadian healthy infant longitudinal development birth cohort study: biological samples and biobanking. Paediatr Perinat Epidemiol. (2015) 29:84–92. doi: 10.1111/ppe.12161, PMID: 25405552

[25] Ewaschuk JB Unger S Connor DLO Stone D Harvey S Clandinin MT. Effect of pasteurization on selected immune components of donated human breast milk. J Perinatol. (2011) 31:593–8. doi: 10.1038/jp.2010.209, PMID: 21330996

[26] Jensen CL Maude M Anderson RE Heird WC. Effect of docosahexaenoic acid supplementation of lactating women on the fatty acid composition of breast milk lipids and maternal and infant plasma phospholipids 1 – 4. Am J Clin Nutr. (2000) 71:292S–9S. doi: 10.1093/ajcn/71.1.292S 10617985

[27] Munhoz J Wattar N Goruk S Pakseresht M Jarman M Forbes L. Determinants of maternal and infant omega-3 status at 3 months postpartum: findings from the APrON longitudinal cohort study. Am J Clin Nutr. (2025) 121:629–42. doi: 10.1016/j.ajcnut.2025.01.002, PMID: 39788297 PMC11923373

[28] Miliku K Duan QL Moraes TJ Becker AB Mandhane PJ Turvey SE. Human milk fatty acid composition is associated with dietary, genetic, sociodemographic, and environmental factors in the CHILD cohort study. Am J Clin Nutr. (2019) 110:1370–83. doi: 10.1093/ajcn/nqz229, PMID: 31589250 PMC6885479

[29] Azad M Robertson B Atakora F Azad MB Becker AB Subbarao P. Human milk oligosaccharide concentrations are associated with multiple fixed and modifiable maternal characteristics, environmental factors, and feeding practices. J Nutr. (2018) 148:1733–42. doi: 10.1093/jn/nxy175, PMID: 30247646

[30] Bayley N. Bayley scales of infant and toddler development–third edition. J Psychoeduc Assess. (2007) 25:180–90. doi: 10.1177/0734282906297199

[31] Bode L. Human milk oligosaccharides: every baby needs a sugar mama. Glycobiology. (2012) 22:1147–62. doi: 10.1093/glycob/cws074, PMID: 22513036 PMC3406618

[32] Hochberg B. Controlling the false discovery rate: a practical and powerful approach to multiple testing. Biometrika. (1995) 61:1–15. doi: 10.2307/2346101

[33] Guxens M Mendez MA Moltó-Puigmartí C Julvez J García-Esteban R Forns J. Breastfeeding, long-chain polyunsaturated fatty cids in colostrum, and infant mental development. Pediatrics. (2011) 128:e880–9. doi: 10.1542/peds.2010-1633, PMID: 21930546 PMC9923846

[34] Beyerlein A Hadders-algra M Kennedy K Fewtrell M Singhal A Rosenfeld E. Infant formula supplementation with long-chain polyunsaturated fatty acids has no effect on Bayley developmental scores at 18 months of age-IPD meta-analysis of 4 large clinical trials. J Pediatr Gastroenterol Nutr. (2010) 50:79–84. doi: 10.1097/MPG.0b013e3181acae7d, PMID: 19881391

[35] Gale CR O’Callaghan FJ Bredow M Martyn CN. The influence of head growth in fetal life, infancy, and childhood on intelligence at the ages of 4 and 8 years. Pediatrics. (2006) 118:1486–92. doi: 10.1542/peds.2005-2629 17015539

[36] Becker M Fehr K Goguen S Miliku K Field C Robertson B. Multimodal machine learning for modeling infant head circumference, mothers’ milk composition, and their shared environment. Sci Rep. (2024) 14:2977–18. doi: 10.1038/s41598-024-52323-w, PMID: 38316895 PMC10844250

[37] Jollife IT Cadima J. Principal component analysis: a review and recent developments. Philos Trans R Soc Lond A Math Phys Eng Sci. (2016) 374:1–16. doi: 10.1098/rsta.2015.0202 PMC4792409 26953178

[38] Zhang Z Wang Y Yang X Cheng Y Zhang H Xu X. Human milk lipid profiles around the world: a systematic review and meta-analysis. Adv Nutr. (2022) 13:2519–36. doi: 10.1093/advances/nmac097, PMID: 36083999 PMC9776668

[39] Khan N Raine L Drollette E Scudder M Hillman C. The relation of saturated fats and dietary cholesterol to childhood cognitive flexibility. Appetitie. (2015) 93:51–6. doi: 10.1016/j.appet.2015.04.012 PMC4546872 25865659

[40] Eskelinen MH Ngandu T Helkala EL Tuomilehto J Nissinen A Soininen H. Fat intake at midlife and cognitive impairment later in life: a population-based CAIDE study. Int J Geriatr Psychiatry. (2008) 23:741–7. doi: 10.1002/gps.1969, PMID: 18188871

[41] Ferreira ALL Alves-Santos NH Freitas-Costa NC Santos PPT Batalha MA Figueiredo ACC. Associations between human milk oligosaccharides at 1 month and infant development throughout the first year of life in a Brazilian cohort. J Nutr. (2021) 151:3543–54. doi: 10.1093/jn/nxab271, PMID: 34313768

[42] Sato K Nakamura Y Fujiyama K Ohneda K Nobukuni T Ogishima S. Absolute quantification of eight human milk oligosaccharides in breast milk to evaluate their concentration profiles and associations with infants’ neurodevelopmental outcomes. J Food Sci. (2024) 89:10152–70. doi: 10.1111/1750-3841.17597, PMID: 39656795 PMC11673463

[43] Zhang S Li T Xie J Zhang D Pi C Zhou L. Gold standard for nutrition: a review of human milk oligosaccharide and its effects on infant gut microbiota. Microb Cell Factories. (2021) 20:108–16. doi: 10.1186/s12934-021-01599-y, PMID: 34049536 PMC8162007

[44] Wang M Li M Wu S Lebrilla CB Chapkin RS Ivanov I. Fecal microbiota composition of breast-fed infants is correlated with human milk oligosaccharides consumed. J Pediatr Gastroenterol Nutr. (2015) 60:825–33. doi: 10.1097/MPG.0000000000000752, PMID: 25651488 PMC4441539

[45] Rose G. Sick individuals and sick populations. Int J Epidemiol. (2001) 30:427–32. doi: 10.1093/ije/30.3.427 11416056

[46] Government of Canada. (2024). Canada’s breastfeeding dashboard. Available online at: https://health-infobase.canada.ca/breastfeeding/. (Accessed March 5, 2025)

[47] Koutra K Chatzi L Roumeliotaki T Vassilaki M Giannakopoulou E Batsos C. Socio-demographic determinants of infant neurodevelopment at 18 months of age: mother–child cohort (Rhea study) in Crete, Greece. Infant Behav Dev. (2012) 35:48–59. doi: 10.1016/j.infbeh.2011.09.005, PMID: 22018719

[48] Cheatham CL Sheppard KW. Synergistic effects of human milk nutrients in the support of infant recognition memory: an observational study. Nutrients. (2015) 7:9079–95. doi: 10.3390/nu7115452, PMID: 26540073 PMC4663580

[49] Li T Samuel TM Zhu Z Howell B Cho S Baluyot K. Joint analyses of human milk fatty acids, phospholipids, and choline in association with cognition and temperament traits during the first 6 months of life. Front Nutr. (2022) 9:1–17. doi: 10.3389/fnut.2022.919769 PMC9449418 36091236

[50] Lewis ZT Totten SM Smilowitz JT Popovic M Parker E Lemay DG. Maternal fucosyltransferase 2 status affects the gut bifidobacterial communities of breastfed infants. Microbiome. (2015) 3:13–7. doi: 10.1186/s40168-015-0071-z, PMID: 25922665 PMC4412032

[51] Berger PK Hampson HE Schmidt KA Alderete TL Furst A Yonemitsu C. Stability of human-milk oligosaccharide concentrations over 1 week of lactation and over 6 hours following a standard meal. J Nutr. (2022) 152:2727–33. doi: 10.1093/jn/nxac214, PMID: 36111739 PMC9839992

[52] Chan K Labonté JM Francis J Zora H Sawchuk S Whitfield KC. Breastfeeding in Canada: predictors of initiation, exclusivity, and continuation from the 2017–2018 Canadian community health survey. Appl Physiol Nutr Metab. (2023) 48:256–69. doi: 10.1139/apnm-2022-0333, PMID: 36596236

