# Supplementary material for: Examining associations between human milk fatty acids, oligosaccharides, and early infant cognitive, language and motor development in the CHILD cohort study
Source: Front Nutr. 2025 Oct 1;12:1606169. doi: 10.3389/fnut.2025.1606169 (PMC12520911; doi:10.3389/fnut.2025.1606169)
Supplement: Supplementary file 1 [file Image_1.pdf]

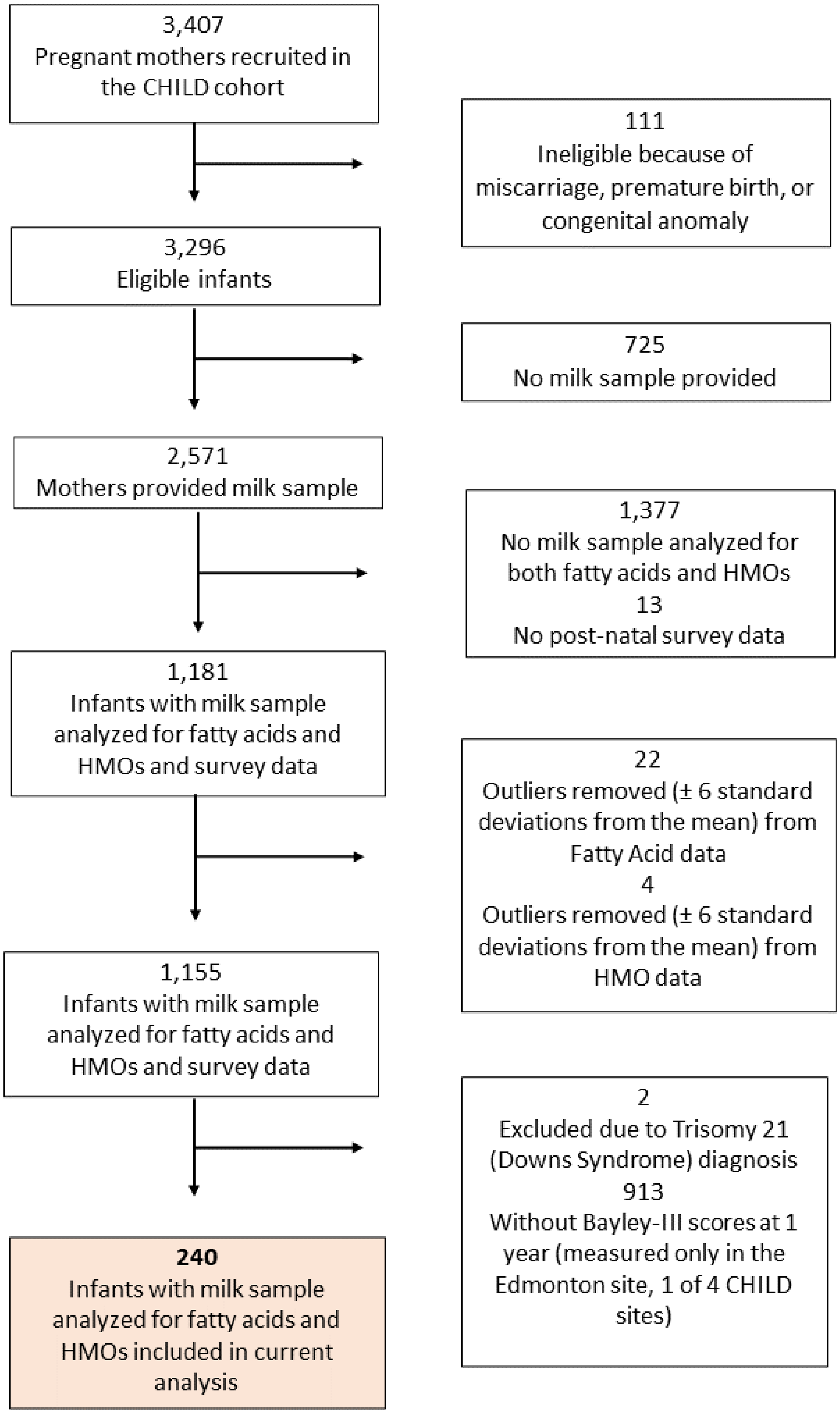

Figure S1: Flow Diagram of CHILD Cohort Study Data used in the Current Analysis

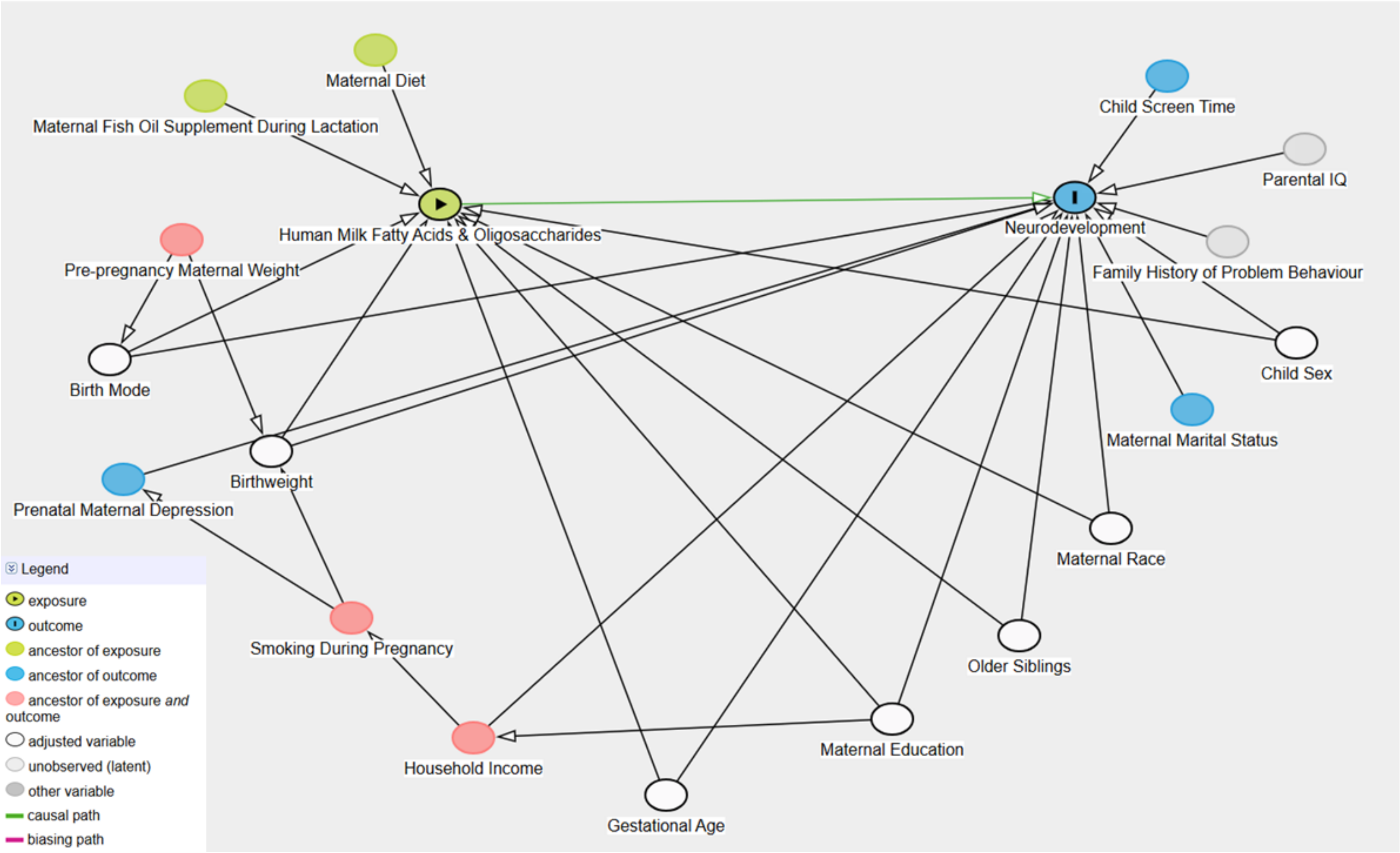

Figure S2: Directed Acyclic Graph to Visualize Relationships Between Variables Included in the Current Study

(A)

Group	Fatty acid	Cognitive 1 Year	Language 1 Year	Motor 1 Year	Cognitive 2 Year	Language 2 Year	Motor 2 Year
Saturated Fatty Acids	Capric Acid (10:0)	0.01	0.02	0.13	-0.01	-0.01	0.04
	Lauric Acid (12:0)	0.04	0.05	0.06	-0.00	0.02	0.01
	Myristic Acid (14:0)	0.08	0.04	0.13	0.03	0.04	0.00
	Palmitic Acid (16:0)	0.15	0.07	0.19	0.08	0.05	0.07
	Margaric Acid (17:0)	0.07	0.01	0.20	0.08	0.09	-0.03
	Stearic Acid (18:0)	0.02	0.03	0.13	0.20	0.18	0.12
Mono-unsaturated Fatty Acids	Palmitoleic Acid (16:ln-7)	0.07	0.08	0.10	-0.07	-0.06	0.02
	Oleic Acid (18:ln-9)	-0.02	-0.07	-0.16	-0.04	-0.03	-0.00
	Vaccenic Acid (18:1 c-11)	-0.03	-0.04	-0.02	0.02	-0.01	0.01
n-3 PUFAs	Alpha Linolenic Acid (18:3n-3)	-0.10	-0.05	-0.11	-0.10	-0.11	-0.11
	Eicosatetraenoic Acid (20:4n-3)	-0.00	-0.01	0.04	-0.02	-0.04	-0.08
	Eicosapentaenoic Acid (20:5n-3)	-0.18	-0.13	-0.17	-0.07	-0.10	-0.10
	Docosapentaenoic Acid (22:5n-3)	-0.20	-0.13	-0.17	-0.10	-0.08	-0.09
	Docosahexaenoic Acid (22:6n-3)	-0.24	-0.12	-0.14	-0.05	-0.06	0.01
	Total n-3	-0.15	-0.08	-0.14	-0.10	-0.12	-0.12
	All n-3 without ALA	-0.21	-0.13	-0.15	-0.07	-0.08	-0.06
n-6 PUFAs	Linoleic Acid (18:2n-6)	-0.20	-0.09	-0.22	-0.10	-0.11	-0.07
	Gamma Linoleic Acid (18:3n-6)	0.04	-0.02	-0.06	-0.07	-0.08	-0.13
	Conjugated Linoleic Acid (18:2c-9, t-11)	-0.01	0.03	0.01	0.02	0.02	-0.08
	Dihomo Gamma Linolenic Acid (20:3n-6)	0.01	-0.06	-0.10	-0.00	0.02	-0.09
	Arachidonic Acid (20:4n-6)	-0.12	-0.11	-0.19	-0.13	-0.02	-0.08
	Adrenic Acid (22:4n-6)	-0.00	-0.08	-0.18	-0.04	0.04	-0.07
	Total n-6	-0.20	-0.10	-0.22	-0.10	-0.11	-0.08
	All n-6 without LA	-0.05	-0.07	-0.12	-0.08	-0.02	-0.12
Fatty Acid Ratios	ARA/DHA+EPA	0.07	0.02	-0.01	-0.03	0.06	0.00
	ARA/DHA	0.06	-0.01	-0.01	-0.05	-0.00	-0.07
	total n-6/total n-3	-0.02	-0.01	-0.02	0.02	0.04	0.09
	LA/ALA	-0.04	-0.02	-0.02	0.02	0.04	0.09
	EPA+DPA/DHA	0.05	-0.03	-0.03	-0.04	-0.06	-0.15

(B)

Group	HMO	Cognitive 1 Year	Language 1 Year	Motor 1 Year	Cognitive 2 Year	Language 2 Year	Motor 2 Year
Fuc+/Sia+	FDSLNH	0.10	0.02	0.08	0.10	0.13	0.04
Fuc+/Sia-	2'FL	0.02	0.06	0.03	-0.06	-0.02	-0.07
	3FL	0.04	0.11	0.10	-0.10	-0.04	-0.02
	DFLac	0.02	0.08	0.07	-0.02	-0.02	-0.00
	LNFP I	-0.06	-0.08	-0.01	-0.13	-0.16	-0.16
	LNFP II	0.06	-0.01	0.12	0.09	0.06	0.08
	LNFP III	-0.07	0.03	0.12	0.07	0.04	0.09
	DFLNT	0.04	0.13	0.12	-0.09	-0.02	-0.02
	FLNH	0.07	0.03	0.09	-0.11	-0.09	-0.11
	DFLNH	-0.05	-0.06	0.03	-0.02	-0.08	-0.09
Fuc-/Sia+	3'SL	0.01	0.12	0.07	-0.10	0.03	0.01
	6'SL	-0.02	0.03	-0.01	-0.07	-0.06	-0.07
	LSTb	-0.07	-0.17	-0.05	0.02	-0.06	-0.04
	LSTc	-0.06	-0.04	-0.03	-0.03	-0.04	-0.08
	DSLNT	-0.12	-0.22	-0.26	-0.08	-0.04	-0.04
	DSLNH	-0.04	-0.04	-0.03	0.01	0.03	0.01
Fuc-/Sia-	LNnT	0.03	-0.02	-0.07	0.07	0.03	-0.01
	LNT	-0.03	-0.13	0.03	-0.01	-0.15	-0.05
	LNH	0.17	0.05	0.09	-0.01	0.08	0.02
Summary Measures	Sialic Acid	0.02	-0.04	0.00	-0.00	0.06	0.04
	Fucose	0.03	0.08	0.11	-0.05	-0.04	-0.05
	Total HMO	0.02	0.01	0.11	-0.03	-0.05	-0.03

Figure S3: Pearson Correlation Coefficients between Human Milk Fatty Acids (A) and HMOs (B) and Bayley-III Scores at 1 and 2 years of Age in the CHILD Cohort Study

Notes: Bayley-III= Bayley Scales of Infant and Toddler Development; DFLac= difucosyllactose; DFLNH= difucosyllacto-N-hexaose; DFLNT= difucosyllacto-N-tetrose; DSLNH= disialyllacto- N-hexaose; DSLNT= disialyllacto-N-tetraose; FLNH= fucosyllacto-N-hexaose; FDSLNH= fucodisialyllacto-N-hexaose; Fuc= Fucosylated HMO; Fucose= human milk oligosaccharide–bound fucose; HMO= human milk oligosaccharide; LNFP I/II/III= lacto-N-fucopentaose-I/II/III; LNH= lacto-N-hexaose; LNnT= lacto-N-neotetraose; LNT= lacto-N-tetrose; LSTb/c= sialyllacto-N-tetraose b/c; Sia= Sialylated HMO; Sialic Acid= HMO-bound Sialic Acid; 2'FL= 2'-fucosyllactose; 3FL= 3-fucosyllactose; 3'SL= 3'-sialyllactose; 6'SL= 6'-sialyllactose; PUFA= Polyunsaturated Fatty Acid; ALA= Alpha Linolenic Acid ; EPA= Eicosapentaenoic Acid; DPA= Docosapentaenoic Acid; DHA= Docosahexaenoic Acid; LA= Linoleic Acid; ARA= Arachidonic Acid. All milk components are expressed as z-scores aside from fatty acid ratios which are not z-scored.

Related to main Figure 1. Correlation coefficients range from -1 to +1; larger coefficients indicate a stronger correlation between the milk component and Bayley-III scores. Red highlight indicates FDR corrected p-value ≤0.05.

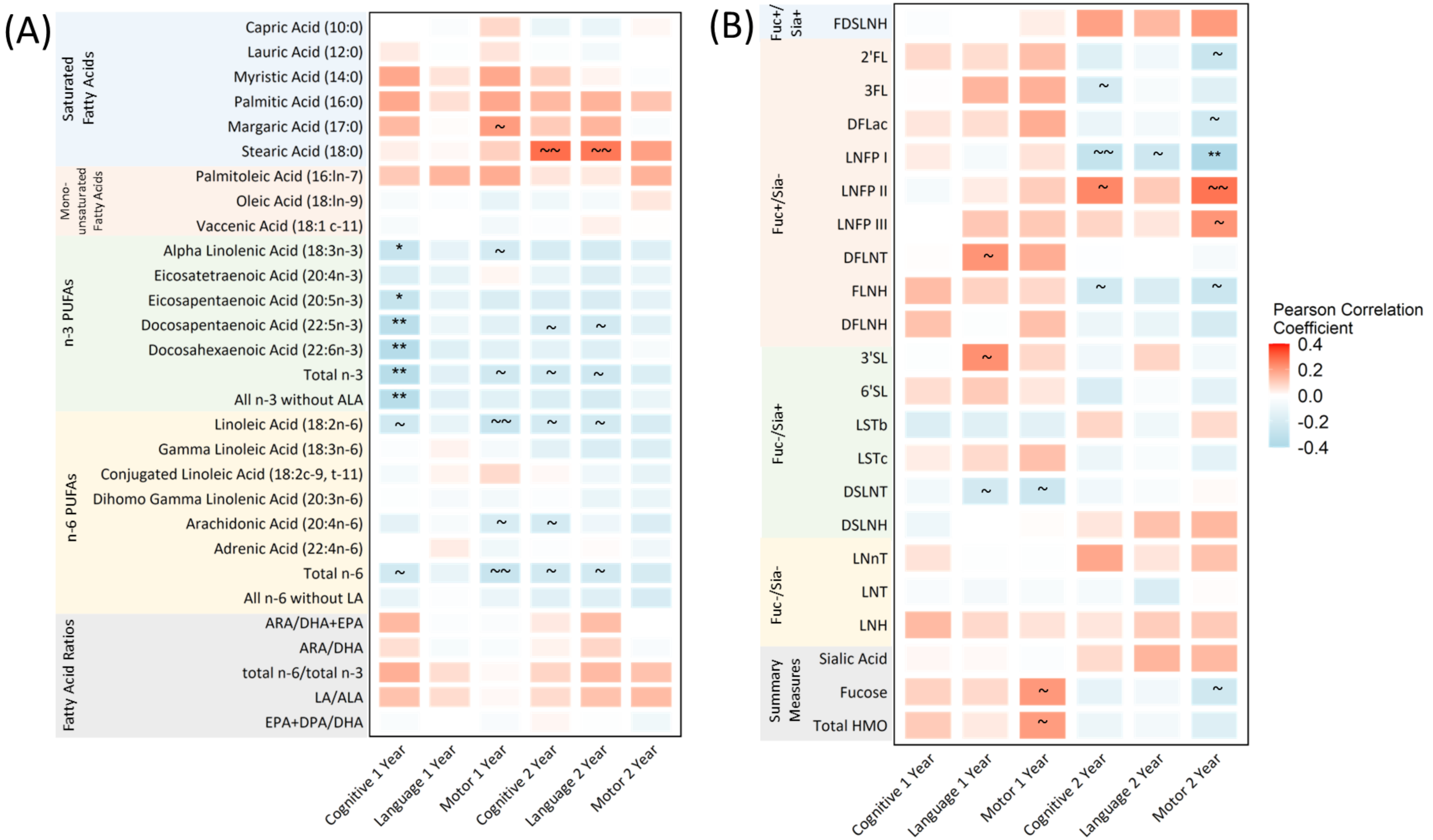

Figure S4: Pearson Correlations between (A) Breastmilk Fatty Acids, (B) HMOs and Bayley-III Scores at 1 and 2 Years of Age in the CHILD Cohort Study among those being Exclusively Breastfed at time of Milk Sampling (n=103)

Notes: Bayley-III= Bayley Scales of Infant and Toddler Development; DFLac= difucosyllactose; DFLNH= difucosyllacto-N-hexaose; DFLNT= difucosyllacto-N-tetrose; DSLNH= disialyllacto- N-hexaose; DSLNT= disialyllacto-N-tetraose; FLNH= fucosyllacto-N-hexaose; FDSLNH= fucodisialyllacto-N-hexaose; Fuc= Fucosylated HMO; Fucose= human milk oligosaccharide–bound fucose; HMO= human milk oligosaccharide; LNFP I/II/III= lacto-N-fucopentaose-I/II/III; LNH= lacto-N-hexaose; LNnT= lacto-N-neotetraose; LNT= lacto-N-tetrose; LSTb/c= sialyllacto-N-tetraose b/c; Sia= Sialylated HMO; Sialic Acid= HMO-bound Sialic Acid; 2’FL= 2’-fucosyllactose; 3FL= 3-fucosyllactose; 3’SL= 3’-sialyllactose; 6’SL= 6’-sialyllactose; PUFA= Polyunsaturated Fatty Acid; ALA= Alpha Linolenic Acid ; EPA= Eicosapentaenoic Acid; DPA= Docosapentaenoic Acid; DHA= Docosahexaenoic Acid; LA= Linoleic Acid; ARA= Arachidonic Acid. Fuc= Fucosylated HMO, Sia= Sialylated HMO. All milk components are expressed at z-scores aside from fatty acid ratios which are not z-scored.

Related to main Figure 1, excluding infants being partially breastfed at time of milk sampling.
*FDR corrected p-value ≤0.05, ** FDR corrected p-value ≤0.01, ~ uncorrected p-value ≤0.05, ~~ uncorrected p-values ≤0.01

(A)

		Cognitive 1 Year	Language 1 Year	Motor 1 Year	Cognitive 2 Year	Language 2 Year	Motor 2 Year
Saturated Fatty Acids	Capric Acid (10:0)	-0.00	-0.03	0.08	-0.11	-0.10	0.02
	Lauric Acid (12:0)	0.04	-0.01	0.06	-0.02	-0.06	-0.01
	Myristic Acid (14:0)	0.18	0.06	0.18	0.10	0.02	-0.02
	Palmitic Acid (16:0)	0.18	0.06	0.18	0.15	0.16	0.12
	Margaric Acid (17:0)	0.15	0.01	0.20	0.11	0.15	-0.03
	Stearic Acid (18:0)	0.03	0.01	0.10	0.29	0.27	0.20
Mono-unsaturated Fatty Acids	Palmitoleic Acid (16:ln-7)	0.11	0.15	0.17	0.05	0.05	0.16
	Oleic Acid (18:ln-9)	-0.04	-0.02	-0.11	-0.07	-0.05	0.05
	Vaccenic Acid (18:1 c-11)	-0.04	-0.00	-0.06	-0.02	0.03	0.00
n-3 PUFAs	Alpha Linolenic Acid (18:3n-3)	-0.28	-0.12	-0.21	-0.20	-0.21	-0.17
	Eicosatetraenoic Acid (20:4n-3)	-0.18	-0.12	0.02	-0.12	-0.17	-0.11
	Eicosapentaenoic Acid (20:5n-3)	-0.28	-0.12	-0.18	-0.18	-0.19	-0.13
	Docosapentaenoic Acid (22:5n-3)	-0.33	-0.10	-0.14	-0.21	-0.22	-0.15
	Docosahexaenoic Acid (22:6n-3)	-0.36	-0.16	-0.14	-0.13	-0.16	-0.04
	Total n-3	-0.35	-0.15	-0.23	-0.23	-0.24	-0.18
	All n-3 without ALA	-0.35	-0.15	-0.15	-0.18	-0.20	-0.11
n-6 PUFAs	Linoleic Acid (18:2n-6)	-0.23	-0.11	-0.27	-0.21	-0.22	-0.19
	Gamma Linoleic Acid (18:3n-6)	-0.02	0.02	-0.05	-0.14	-0.18	-0.18
	Conjugated Linoleic Acid (18:2c-9, t-11)	-0.06	0.02	0.08	0.01	-0.08	-0.12
	Dihomo Gamma Linolenic Acid (20:3n-6)	-0.01	-0.05	-0.06	-0.02	-0.11	-0.10
	Arachidonic Acid (20:4n-6)	-0.13	-0.04	-0.20	-0.21	-0.09	-0.18
	Adrenic Acid (22:4n-6)	-0.00	0.04	-0.07	-0.02	0.01	-0.08
	Total n-6	-0.23	-0.11	-0.27	-0.22	-0.22	-0.20
	All n-6 without LA	-0.11	-0.02	-0.10	-0.15	-0.16	-0.20
Fatty Acid Ratios	ARA/DHA+EPA	0.15	-0.02	-0.03	0.05	0.14	0.00
	ARA/DHA	0.07	-0.04	-0.03	0.03	0.09	-0.04
	total n-6/total n-3	0.16	0.07	0.01	0.09	0.14	0.13
	LA/ALA	0.13	0.07	0.01	0.08	0.13	0.14
	EPA+DPA/DHA	-0.03	0.00	-0.04	0.02	-0.01	-0.07

(B)

Group	HMO	Cognitive 1 Year	Language 1 Year	Motor 1 Year	Cognitive 2 Year	Language 2 Year	Motor 2 Year
Fuc+/Sia+	FDSLNH	-0.02	0.00	0.04	0.19	0.15	0.21
Fuc+/Sia-	2'FL	0.08	0.07	0.13	-0.16	-0.07	-0.27
	3FL	0.00	0.15	0.16	-0.21	-0.04	-0.15
	DFLac	0.05	0.07	0.17	-0.10	-0.06	-0.23
	LNFP I	0.04	-0.05	0.06	-0.29	-0.24	-0.39
	LNFP II	-0.05	0.04	0.10	0.24	0.11	0.28
	LNFP III	-0.01	0.12	0.11	0.09	0.05	0.22
	DFLNT	0.00	0.22	0.17	-0.01	0.00	-0.05
	FLNH	0.14	0.09	0.08	-0.23	-0.18	-0.26
	DFLNH	0.13	-0.01	0.13	-0.09	-0.11	-0.20
Fuc-/Sia+	3'SL	-0.01	0.23	0.08	-0.07	0.09	-0.06
	6'SL	0.07	0.11	0.05	-0.18	-0.03	-0.13
	LSTb	-0.17	-0.16	-0.14	0.09	-0.08	0.08
	LSTc	0.04	0.08	0.13	-0.09	-0.03	-0.13
	DSLNT	-0.06	-0.22	-0.25	-0.07	-0.03	0.01
	DSLNH	-0.08	-0.00	0.01	0.05	0.13	0.15
Fuc-/Sia-	LNnT	0.06	-0.01	-0.01	0.18	0.05	0.13
	LNT	-0.04	-0.06	-0.04	-0.05	-0.18	0.01
	LNH	0.14	0.08	0.06	0.05	0.10	0.11
Summary Measures	Sialic Acid	0.02	0.01	-0.02	0.08	0.15	0.15
	Fucose	0.09	0.08	0.21	-0.12	-0.07	-0.23
	Total HMO	0.11	0.04	0.20	-0.09	-0.06	-0.16

Figure S5: Pearson Correlation Coefficients between Human Milk Fatty acids (A) and HMOs (B) and Bayley-III Scores at 1 and 2 years of Age in the CHILD Cohort Study among those being Exclusively Breastfed at time of Milk Sampling (n=103)

Notes: Bayley-III= Bayley Scales of Infant and Toddler Development; DFLac= difucosyllactose; DFLNH= difucosyllacto-N-hexaose; DFLNT= difucosyllacto-N-tetrose; DSLNH= disialyllacto- N-hexaose; DSLNT= disialyllacto-N-tetraose; FLNH= fucosyllacto-N-hexaose; FDSLNH= fucodisialyllacto-N-hexaose; Fuc= Fucosylated HMO; Fucose= human milk oligosaccharide–bound fucose; HMO= human milk oligosaccharide; LNFP I/II/III= lacto-N-fucopentaose-I/II/III; LNH= lacto-N-hexaose; LNnT= lacto-N-neotetraose; LNT= lacto-N-tetrose; LSTb/c= sialyllacto-N-tetraose b/c; Sia= Sialylated HMO; Sialic Acid= HMO-bound Sialic Acid; 2'FL= 2'-fucosyllactose; 3FL= 3-fucosyllactose; 3'SL= 3'-sialyllactose; 6'SL= 6'-sialyllactose; PUFA= Polyunsaturated Fatty Acid; ALA= Alpha Linolenic Acid ; EPA= Eicosapentaenoic Acid; DPA= Docosapentaenoic Acid; DHA= Docosahexaenoic Acid; LA= Linoleic Acid; ARA= Arachidonic Acid. All milk components are expressed as z-scores aside from fatty acid ratios which are not z-scored.

Related to Supplementary Figure S4. Correlation coefficients range from -1 to +1; larger coefficients indicate a stronger correlation between the milk component and Bayley-III scores. Red highlight indicates FDR corrected p-value ≤0.05.

(A) Cognitive Score

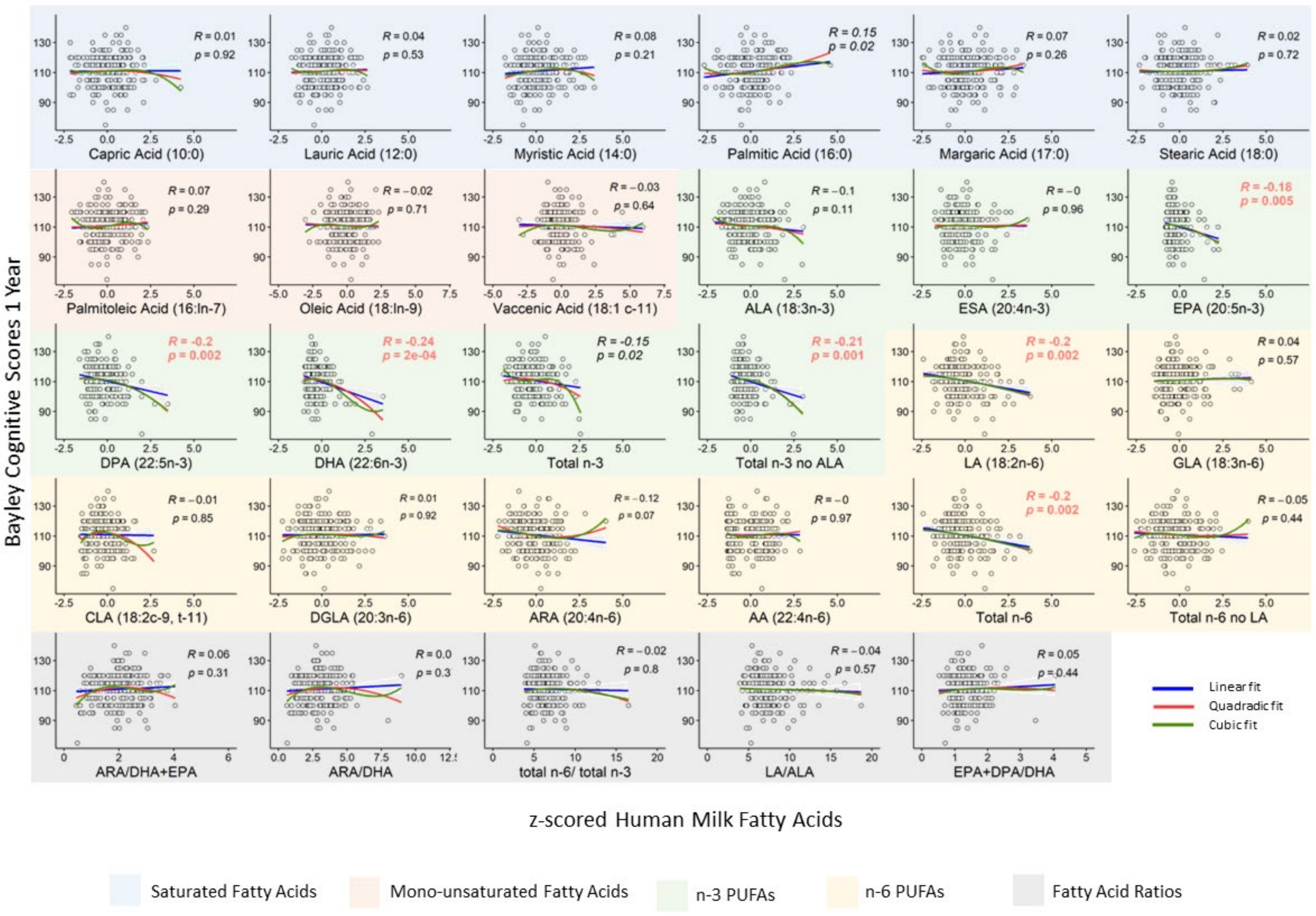

(B) Language Score

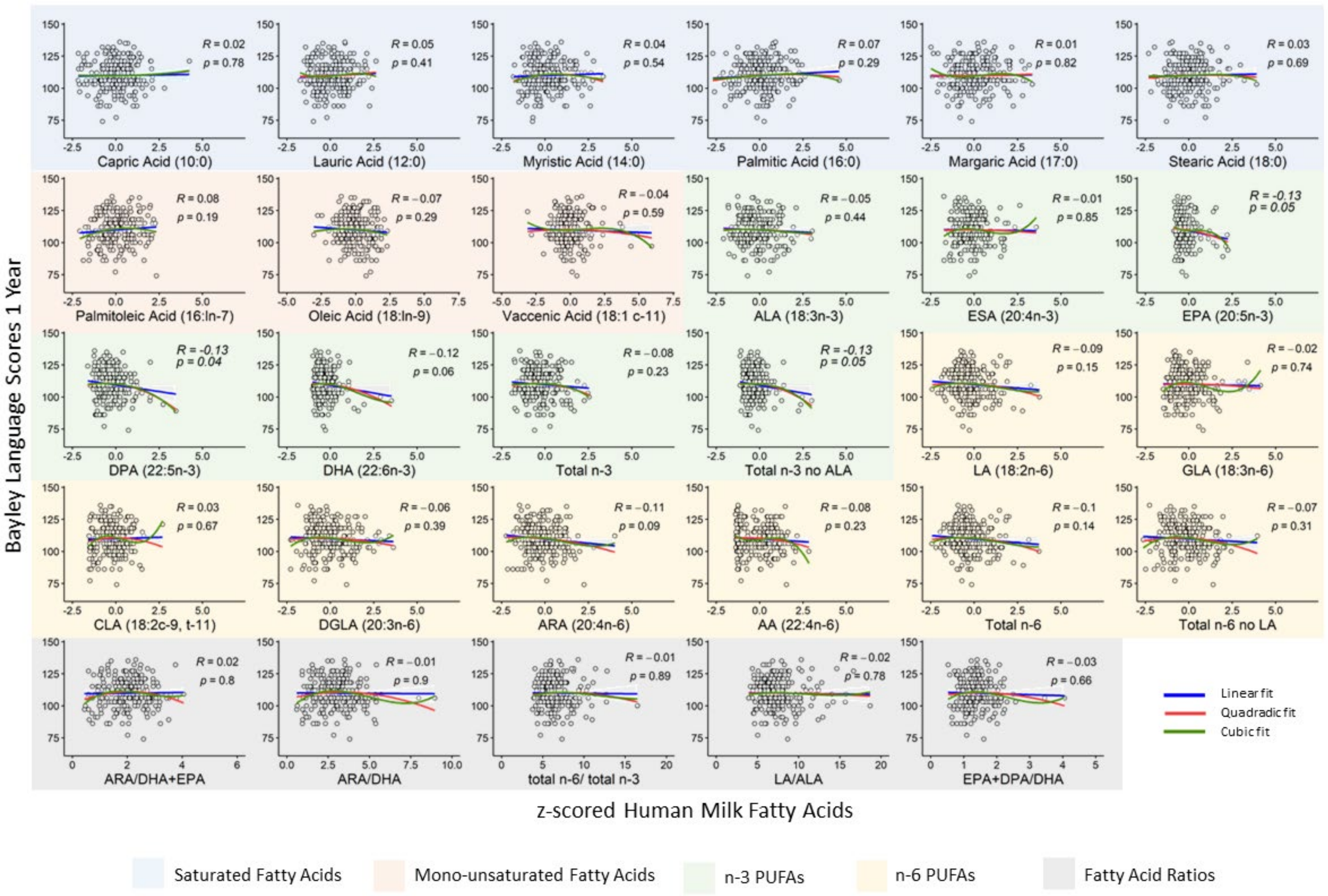

(C) Motor Score

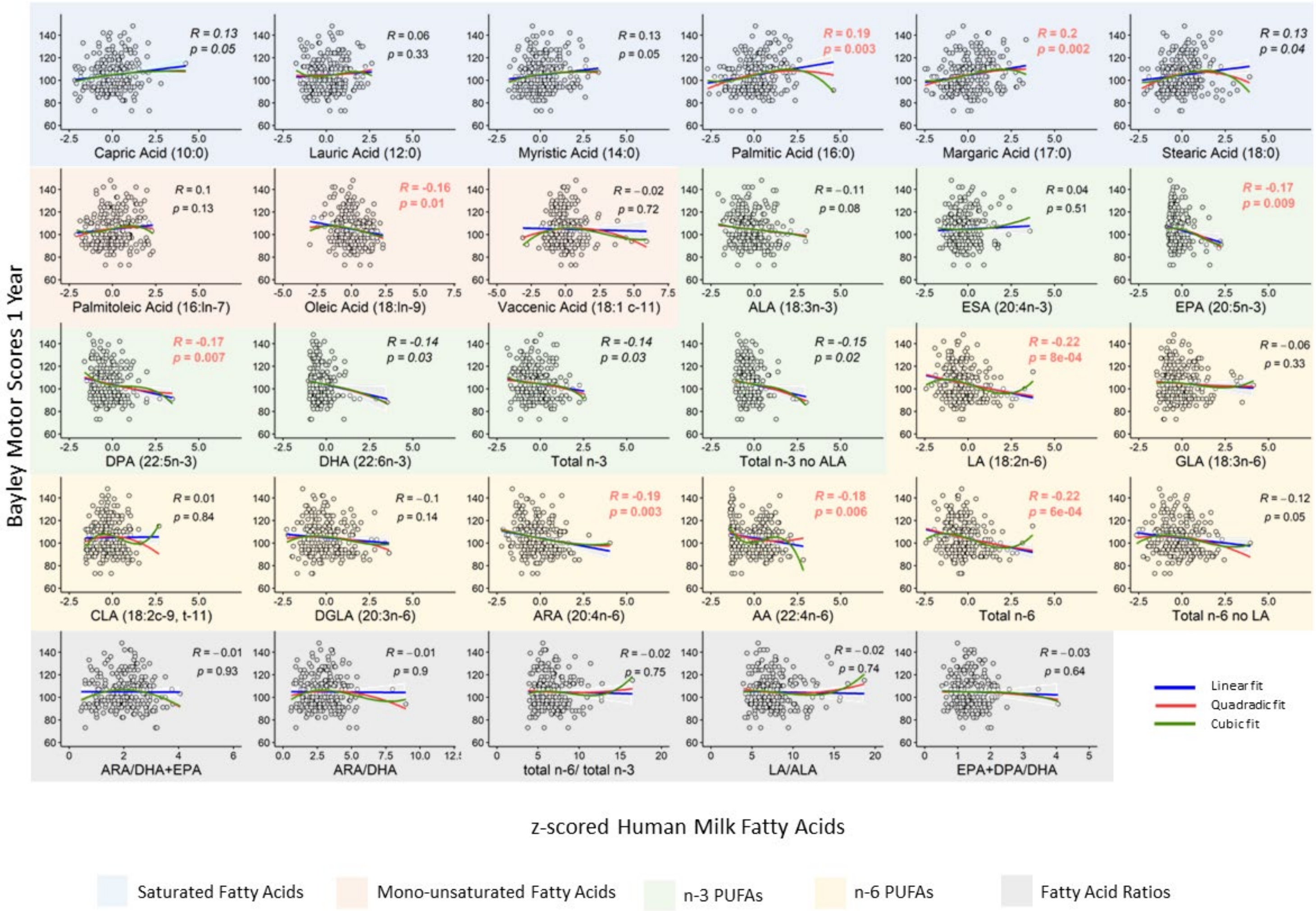

Figure S6: Scatter Plots of Fatty Acids and Bayley-III Scores at One Year in the CHILD Cohort Study

Notes: Bayley-III= Bayley Scales of Infant and Toddler Development; PUFA= Polyunsaturated Fatty Acid; ALA= Alpha Linolenic Acid; ESA= Eicosatetraenoic Acid; EPA= Eicosapentaenoic Acid; DPA= Docosapentaenoic Acid; DHA= Docosahexaenoic Acid; LA= Linoleic Acid; GLA= Gamma Linoleic Acid; CLA=; Conjugated Linoleic Acid; DGLA= Dihomo Gamma Linolenic Acid; ARA= Arachidonic Acid; AA= Adrenic Acid. Fatty acid ratios are not z-scored.

Scatter plots R values align with Pearson correlation coefficients in Supplementary Figure S3. R values indicate unadjusted Pearson correlation coefficient; p-values that remained significant to ≤0.05 after FDR correction are indicated by red text; Uncorrected p-values ≤0.05 are indicated by italicized text.

(A) Cognitive

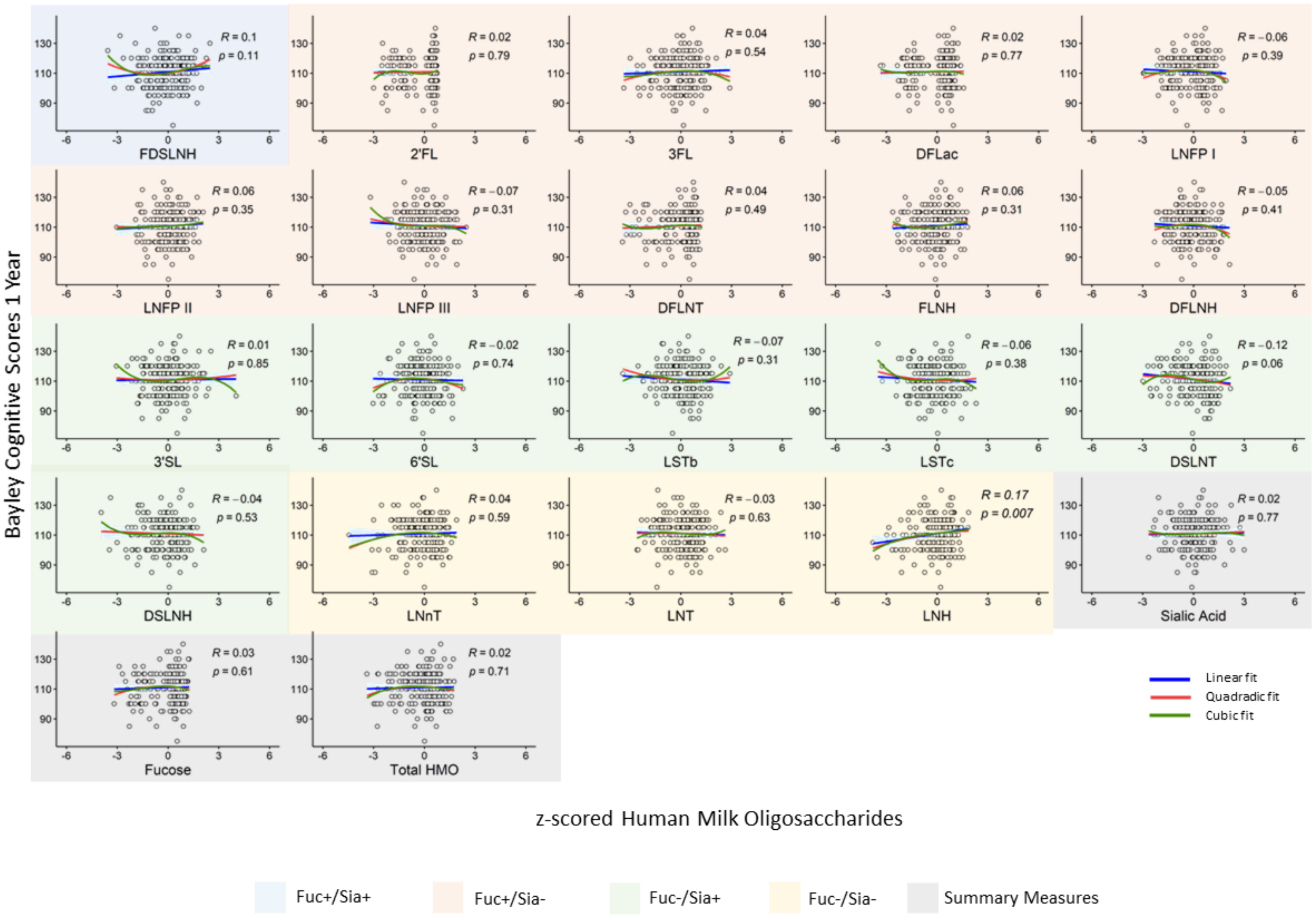

Bayley Cognitive Scores 1 Year
FDSLNH
R = 0.1
p = 0.11
2'FL
R = 0.02
p = 0.79
3FL
R = 0.04
p = 0.54
DFLac
R = 0.02
p = 0.77
LNFP I
R = -0.06
p = 0.39
LNFP II
R = 0.06
p = 0.35
LNFP III
R = -0.07
p = 0.31
DFLNT
R = 0.04
p = 0.49
FLNH
R = 0.06
p = 0.31
DFLNH
R = -0.05
p = 0.41
3'SL
R = 0.01
p = 0.85
6'SL
R = -0.02
p = 0.74
LSTb
R = -0.07
p = 0.31
LSTc
R = -0.06
p = 0.38
DSLNT
R = -0.12
p = 0.06
DSLNH
R = -0.04
p = 0.53
LNnT
R = 0.04
p = 0.59
LNT
R = -0.03
p = 0.63
LNH
R = 0.17
p = 0.007
Sialic Acid
R = 0.02
p = 0.77
Fucose
R = 0.03
p = 0.61
Total HMO
R = 0.02
p = 0.71
Linear fit
Quadradic fit
Cubic fit
z-scored Human Milk Oligosaccharides
Fuc+/Sia+
Fuc+/Sia-
Fuc-/Sia+
Fuc-/Sia-
Summary Measures

(B) Language

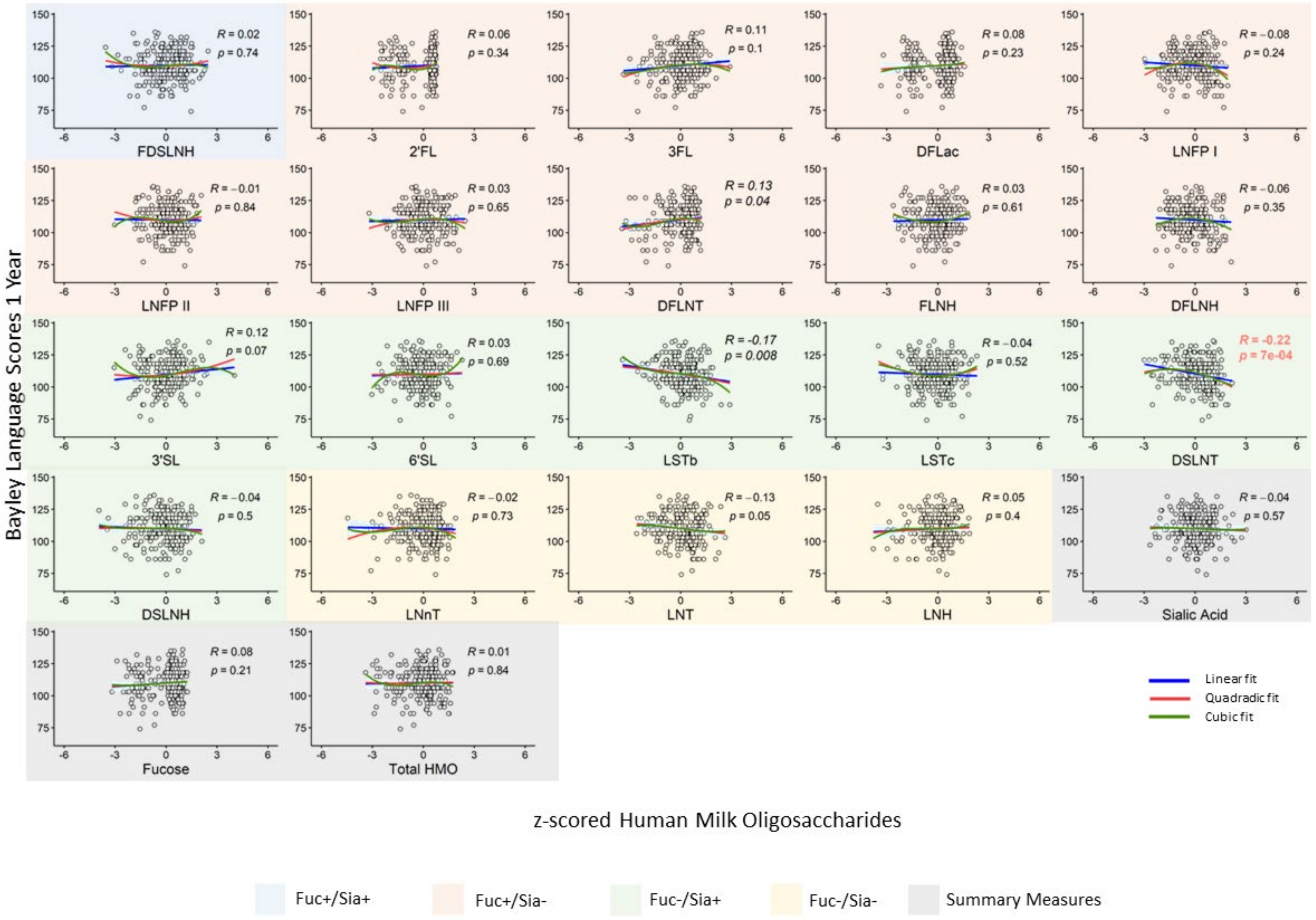
Bayley Language Scores 1 Year
FDSLNH R = 0.02 p = 0.74
2'FL R = 0.06 p = 0.34
3FL R = 0.11 p = 0.1
DFLac R = 0.08 p = 0.23
LNFP I R = −0.08 p = 0.24
LNFP II R = −0.01 p = 0.84
LNFP III R = 0.03 p = 0.65
DFLNT R = 0.13 p = 0.04
FLNH R = 0.03 p = 0.61
DFLNH R = −0.06 p = 0.35
3'SL R = 0.12 p = 0.07
6'SL R = 0.03 p = 0.69
LSTb R = -0.17 p = 0.008
LSTc R = −0.04 p = 0.52
DSLNT R = -0.22 p = 7e-04
DSLNH R = −0.04 p = 0.5
LNnT R = −0.02 p = 0.73
LNT R = −0.13 p = 0.05
LNH R = 0.05 p = 0.4
Sialic Acid R = −0.04 p = 0.57
Fucose R = 0.08 p = 0.21
Total HMO R = 0.01 p = 0.84
Linear fit
Quadradic fit
Cubic fit
z-scored Human Milk Oligosaccharides
Fuc+/Sia+
Fuc+/Sia-
Fuc-/Sia+
Fuc-/Sia-
Summary Measures

(C) Motor

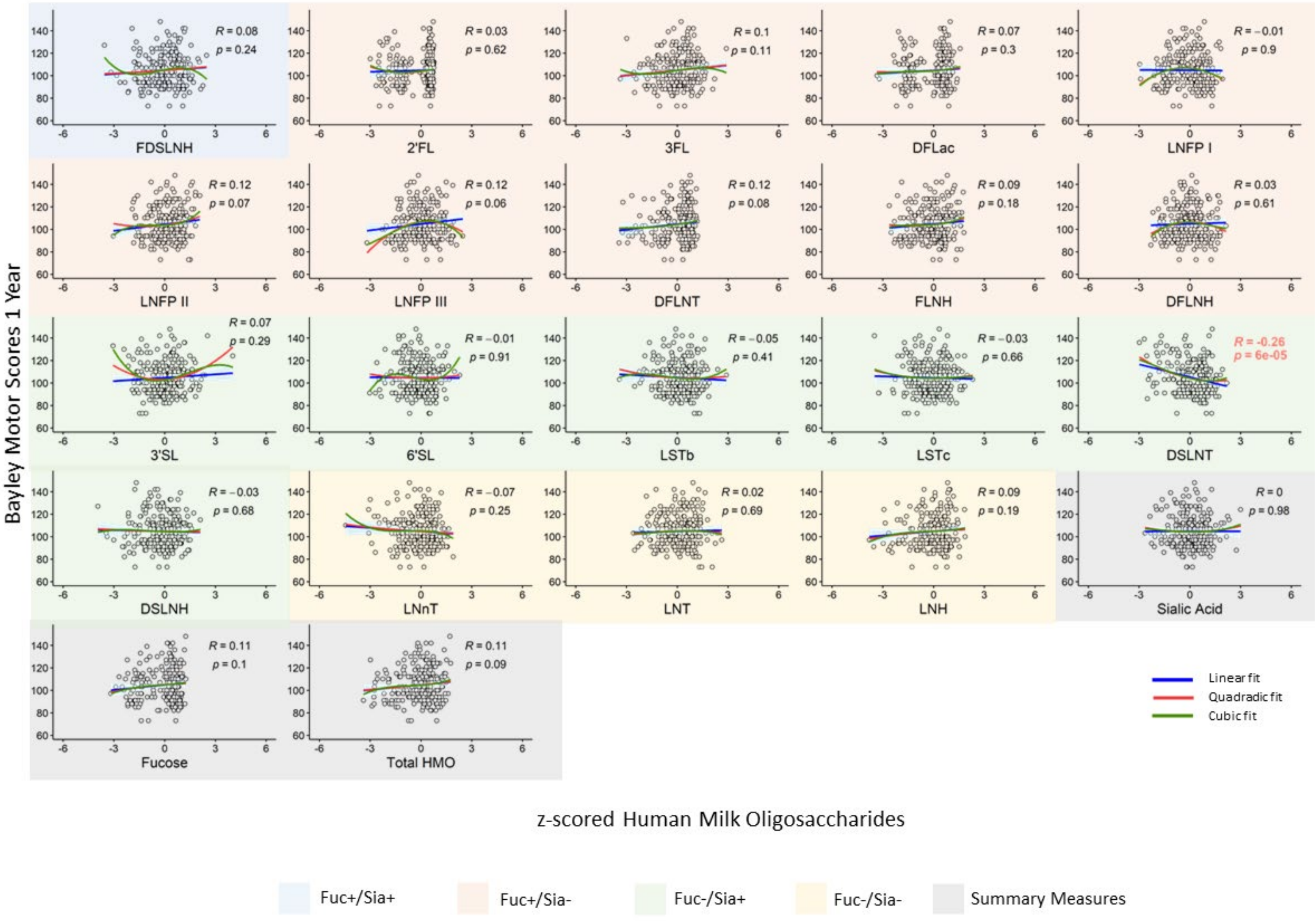

Figure S7: Scatter Plots of HMOs and Bayley-III Scores at One Year in the CHILD Cohort Study

Notes: Bayley-III= Bayley Scales of Infant and Toddler Development; DFLac= difucosyllactose; DFLNH= difucosyllacto-N-hexaose; DFLNT= difucosyllacto-N-tetrose; DSLNH= disialyllacto- N-hexaose; DSLNT= disialyllacto-N-tetraose; FLNH= fucosyllacto-N-hexaose; FDSLNH= fucodisialyllacto-N-hexaose; Fuc= Fucosylated HMO; Fucose= human milk oligosaccharide–bound fucose; HMO= human milk oligosaccharide; LNFP I/II/III= lacto-N-fucopentaose-I/II/III; LNH= lacto-N-hexaose; LNnT= lacto-N-neotetraose; LNT= lacto-N-tetrose; LSTb/c= sialyllacto-N-tetraose b/c; Sia= Sialylated HMO; Sialic Acid= HMO-bound Sialic Acid; 2'FL= 2'-fucosyllactose; 3FL= 3-fucosyllactose; 3'SL= 3'-sialyllactose; 6'SL= 6'-sialyllactose.

Scatter plot R values align with Pearson correlation coefficients in Supplementary Figure S3. R values indicate unadjusted correlation coefficient; p-values that remained significant to ≤0.05 after FDR correction are indicated by red text; Uncorrected p-values ≤0.05 are indicated by italicized text.

Fatty Acids					Human Milk Oligosaccharides			
	Factor Loadings for Principal Component One	Factor Loadings for Principal Component Two	Factor Loadings for Principal Component Three			Factor Loadings for Principal Component One	Factor Loadings for Principal Component Two	Factor Loadings for Principal Component Three
**Saturated Fatty Acids**					**Fuc+/Sia+**			
Capric Acid (10:0)	0.06	0.09	0.40		FDSLNH	-0.28	-0.34	0.04
Lauric Acid (12:0)	0.07	0.20	0.54		**Fuc+/Sia-**			
Myristic Acid (14:0)	0.18	0.30	0.36		2'FL	0.40	0.01	0.07
Palmitic Acid (16:0)	0.27	0.29	-0.25		3FL	0.29	-0.14	-0.19
Margaric Acid (17:0)	0.17	0.30	-0.24		DFLac	0.38	-0.01	0.01
Stearic Acid (18:0)	0.26	0.13	-0.22		LNFP1	0.34	0.25	0.23
**Monounsaturated Fatty Acids**					LNFP11	-0.33	-0.25	0.06
Palmitoleic Acid (16:ln-7)	0.05	0.26	-0.24		LNFP111	-0.22	0.04	0.03
Oleic Acid (18:ln-9)	-0.17	-0.34	-0.13		DFLNT	0.23	-0.24	0.04
Vaccenic Acid (18:1 c-11)	-0.07	-0.05	-0.24		FLNH	0.09	-0.14	0.22
**n-3 PUFAs**					DFLNH	0.17	0.19	0.32
ALA (18:3n-3)	-0.28	-0.20	0.10		**Fuc-/Sia+**			
ESA (20:4n-3)	-0.25	0.27	0.08		3'SL	0.24	-0.25	-0.17
EPA (20:5n-3)	-0.25	0.16	-0.16		6'SL	0.04	-0.20	0.30
DPA (22:5n-3)	-0.32	0.26	-0.14		LSTb	-0.21	0.22	0.25
DHA (22:6n-3)	-0.27	0.11	-0.11		LSTc	0.17	-0.22	0.35
**n-6 PUFAs**					DSLNT	0.02	0.07	0.34
LA (18:2n-6)	-0.24	-0.29	0.11		DSLNH	-0.11	-0.43	0.27
GLA (18:3n-6)	-0.27	0.07	0.00		**Fuc-/Sia-**			
CLA (18:2c-9, t-11)	-0.20	0.23	0.10		LNnT	-0.01	0.14	0.29
DGLA (20:3n-6)	-0.24	0.22	0.11		LNT	-0.18	0.24	0.37
ARA (20:4n-6)	-0.28	0.15	-0.07		LNH	0.06	-0.40	0.18
AA (22:4n-6)	-0.26	0.24	0.00					
**Explained variance (%)**	25.2%	16.9%	11.8%		**Explained variance (%)**	27.2%	13.6%	11.8%

Figure S8: Factor Loadings for Fatty Acid and HMO Principal Components One, Two and Three in the CHILD Cohort Study

Notes: DFLac= difucosyllactose; DFLNH= difucosyllacto-N-hexaose; DFLNT= difucosyllacto-N-tetrose; DSLNH= disialyllacto- N-hexaose; DSLNT= disialyllacto-N-tetraose; FLNH= fucosyllacto-N-hexaose; FDSLNH= fucodisialyllacto-N-hexaose; Fuc= Fucosylated HMO; Fucose= human milk oligosaccharide–bound fucose; HMO= human milk oligosaccharide; LNFP I/II/III= lacto-N-fucopentaose-I/II/III; LNH= lacto-N-hexaose; LNnT= lacto-N-neotetraose; LNT= lacto-N-tetrose; LSTb/c= sialyllacto-N-tetraose b/c; Sia= Sialylated HMO; Sialic Acid= HMO-bound Sialic Acid; 2'FL= 2'-fucosyllactose; 3FL= 3-fucosyllactose; 3'SL= 3'-sialyllactose; 6'SL= 6'-sialyllactose; PUFA= Polyunsaturated Fatty Acid; ALA= Alpha Linolenic Acid; ESA= Eicosatetraenoic Acid; EPA= Eicosapentaenoic Acid; DPA= Docosapentaenoic Acid; DHA= Docosahexaenoic Acid; LA= Linoleic Acid; GLA= Gamma Linoleic Acid; CLA=; Conjugated Linoleic Acid; DGLA= Dihomo Gamma Linolenic Acid; ARA= Arachidonic Acid; AA= Adrenic Acid.

	Bayley-III Scales 1 Year			Bayley-III Scales 2 Years		
	**Cognitive**	**Language**	**Motor**	**Cognitive**	**Language**	**Motor**
**Saturated Fatty Acids**						
Capric Acid (10:0)						
Lauric Acid (12:0)						
Myristic Acid (14:0)						
Palmitic Acid (16:0)						
Margaric Acid (17:0)					^	~~
Stearic Acid (18:0)						
**Monounsaturated Fatty Acids**						
Palmitoleic Acid (16:ln-7)						
Oleic Acid (18:ln-9)						
Vaccenic Acid (18:1 c-11)						
**n-3 PUFAs**						
ALA (18:3n-3)		^	^			
ESA (20:4n-3)						
EPA (20:5n-3)						^
DPA (22:5n-3)						^
DHA (22:6n-3)						~
Total n-3		^	^			
Total n-3 no ALA						~
**n-6 PUFAs**						
LA (18:2n-6)						
GLA (18:3n-6)						
CLA (18:2c-9m t-11)	^					
DGLA (20:3n-6)						
ARA (20:4n-6)						
AA (22:4n-6)	^					
Total n-6						
Total n-6 no LA	^					
**Ratios**						
ARA/ DHA+ EPA						~~
ARA/DHA						~~
Total n-6/ total n-3		^				
LA/ ALA		~				
EPA+DPA/ DHA						

Saturated Fatty Acids Mono-unsaturated Fatty Acids n-3 PUFAs n-6 PUFAs Fatty Acid Ratios

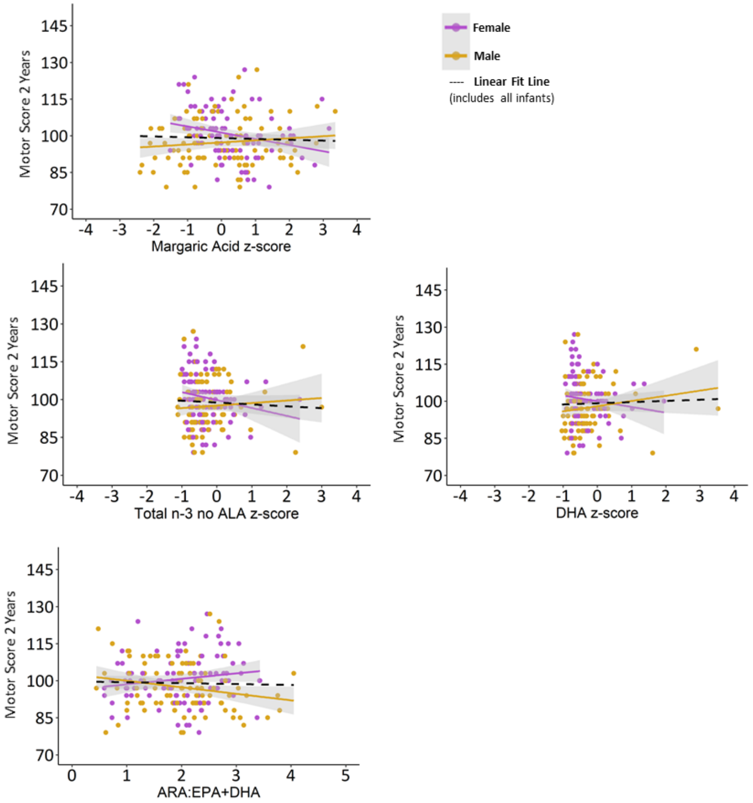

Figure S9: Adjusted Interactions between Sex and Fatty Acids on Bayley-III Scores at One and Two Years in the CHILD Cohort Study

Notes: Bayley-III= Bayley Scales of Infant and Toddler Development; PUFA= Polyunsaturated Fatty Acid; ALA= Alpha Linolenic Acid; ESA= Eicosatetraenoic Acid; EPA= Eicosapentaenoic Acid; DPA= Docosapentaenoic Acid; DHA= Docosahexaenoic Acid; LA= Linoleic Acid; GLA= Gamma Linoleic Acid; CLA=; Conjugated Linoleic Acid; DGLA= Dihomo Gamma Linolenic Acid; ARA= Arachidonic Acid; AA= Adrenic Acid.

Coloured cells in the table are represented as scatter plots. All models are adjusted for: birthweight, birth mode, number of older siblings, gestational age, maternal race, maternal education, infant age at milk sampling. Linear fit line is from a model that does not include the interaction term.

^ uncorrected p-value ≤0.1, ~ uncorrected p-value ≤0.05; ~~uncorrected p-value ≤ 0.01; there are no significant p-values after FDR correction

	Bayley-III Scales 1 Year			Bayley-III Scales 2 Years		
	Cognitive	Language	Motor	Cognitive	Language	Motor
**Fuc+/Sia+**						
FDSLNH					~	
**Fuc+/Sia-**						
2'FL						
3FL			~			
DFLac						^
LNFP1						
LNFP11						
LNFP111						
DFLNT						
FLNH						~
DFLNH					^	~
**Fuc-/Sia+**						
3'SL		^				
6'SL						
LSTb						
LSTc						
DSLNT						
DSLNH		^				
**Fuc-/Sia-**						
LNnT	~	^				
LNT						
LNH						
**Summary Measures**						
Total Sialic Acid						^
Total Fucose						
Total HMO						

Fuc+/Sia+ Fuc+/Sia- Fuc-/Sia+ Fuc-/Sia- Summary Measures

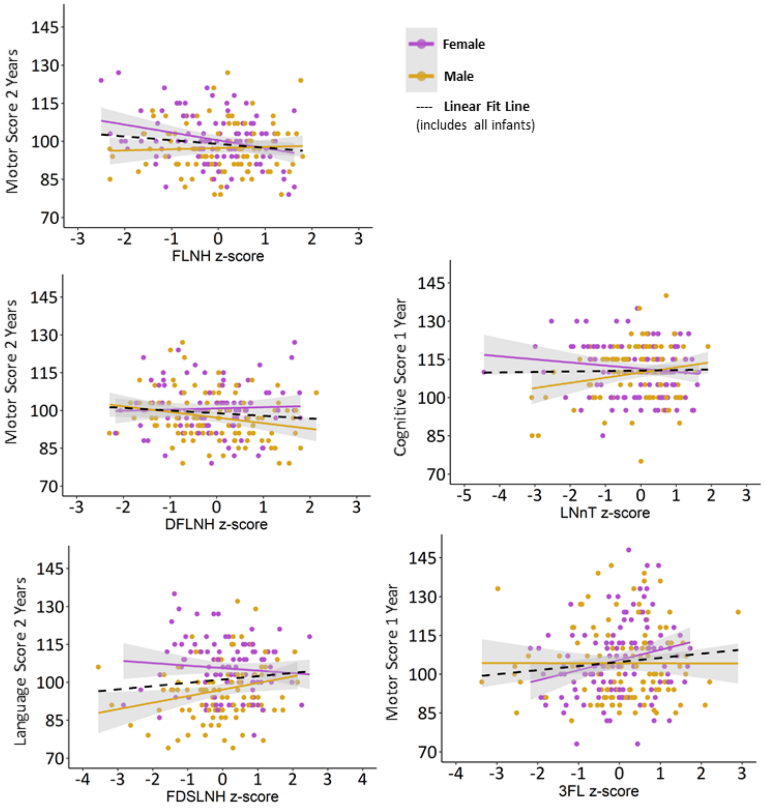

Figure S10: Adjusted Interactions between Sex and HMOs on Bayley-III Scores at One and Two Years in the CHILD Cohort Study

Notes: Bayley-III= Bayley Scales of Infant and Toddler Development; DFLac= difucosyllactose; DFLNH= difucosyllacto-N-hexaose; DFLNT= difucosyllacto-N-tetrose; DSLNH= disialyllacto- N-hexaose; DSLNT= disialyllacto-N-tetraose; FLNH= fucosyllacto-N-hexaose; FDSLNH= fucodisialyllacto-N-hexaose; Fuc= Fucosylated HMO; Fucose= human milk oligosaccharide–bound fucose; HMO= human milk oligosaccharide; LNFP I/II/III= lacto-N-fucopentaose-I/II/III; LNH= lacto-N-hexaose; LNnT= lacto-N-neotetraose; LNT= lacto-N-tetrose; LSTb/c= sialyllacto-N-tetraose b/c; Sia= Sialylated HMO; Sialic Acid= HMO-bound Sialic Acid; 2'FL= 2'-fucosyllactose; 3FL= 3-fucosyllactose; 3'SL= 3'-sialyllactose; 6'SL= 6'-sialyllactose.

Coloured cells in the table are represented as scatter plots. All models are adjusted for: birthweight, birth mode, number of older siblings, gestational age, maternal race, maternal education, infant age at milk sampling. Linear fit line is from a model that does not include the interaction term.

^ uncorrected p-value ≤0.1, ~ uncorrected p-value ≤0.05; there are no significant p-values after FDR correction
